# Prevalence of *Mycoplasma genitalium* in different population groups: systematic review andmeta-analysis

**DOI:** 10.1136/sextrans-2017-053384

**Published:** 2018-02-09

**Authors:** Lukas Baumann, Manuel Cina, Dianne Egli-Gany, Myrofora Goutaki, Florian S Halbeisen, Gian-Reto Lohrer, Hammad Ali, Pippa Scott, Nicola Low

**Affiliations:** 1 Institute of Social and Preventive Medicine, University of Bern, Bern, Switzerland; 2 Kirby Institute, University of New South Wales, Sydney, New South Wales, Australia; 3 Department of Pathology, University of Otago, Christchurch, New Zealand

**Keywords:** systematic rreviews, meta-analysis, epidemiology (general)

## Abstract

**Background:**

*Mycoplasma genitalium* is a common cause of non-gonococcal non-chlamydial urethritis and cervicitis. Testing of asymptomatic populations has been proposed, but prevalence in asymptomatic populations is not well established. We aimed to estimate the prevalence of *M. genitalium* in the general population, pregnant women, men who have sex with men (MSM), commercial sex workers (CSWs) and clinic-based samples.

**Methods:**

We searched Embase, Medline, IndMED, African Index Medicus and LILACS from 1 January 1991 to 12 July 2016 without language restrictions. We included studies with 500 participants or more. Two reviewers independently screened and selected studies and extracted data. We examined forest plots and conducted random-effects meta-analysis to estimate prevalence, if appropriate. Between-study heterogeneity was examined using the I^2^ statistic and meta-regression.

**Results:**

Of 3316 screened records, 63 were included. In randomly selected samples from the general population, the summary prevalence was 1.3% (95% CI 1.0% to 1.8%, I^2^ 41.5%, three studies, 9091 people) in countries with higher levels of development and 3.9% (95% CI 2.2 to 6.7, I^2^ 89.2%, three studies, 3809 people) in countries with lower levels. Prevalence was similar in women and men (P=0.47). In clinic based samples, prevalence estimates were higher, except in asymptomatic patients (0.8%, 95% CI 0.4 to 1.4, I^2^ 0.0%, three studies, 2889 people). Summary prevalence estimates were, in the following groups: pregnant women 0.9% (95% CI 0.6% to 1.4%, I^2^ 0%, four studies, 3472 people), MSM in the community 3.2% (95% CI 2.1 to 5.1, I^2^ 78.3%, five studies, 3012 people) and female CSWs in the community 15.9% (95% CI 13.5 to 18.9, I^2^ 79.9%, four studies, 4006 people).

**Discussion:**

This systematic review can inform testing guidelines for *M. genitalium*. The low estimated prevalence of *M. genitalium* in the general population, pregnant women and asymptomatic attenders at clinics does not support expansion of testing to these groups.

**Registration numbers:**

PROSPERO: CRD42015020420

## Introduction

*Mycoplasma genitalium* is a cause of non-gonococcal non-chlamydial urethritis in men and cervicitis in women,^1-3^ and is reported to be associated with pelvic inflammatory disease, infertility and preterm birth.^4^ *M. genitalium* was first isolated in the early 1980s in men with non-gonococcal urethritis^5^ but, owing to difficulties in detecting the microorganism by culture, most research on *M. genitalium* has been done since the development of nucleic acid amplification tests (NAATs) in the early 1990s.^1^ In populations studied in healthcare settings, *M. genitalium* has been detected in substantial proportions of men with urethritis and women with cervicitis.^1 2^ Based on these studies, routine testing has been suggested to detect and treat *M. genitalium* in asymptomatic attenders in healthcare settings^6^ and the recommendation has also been extended to low risk general populations.^7^ Multiplex NAATs are being used increasingly to detect multiple sexually transmitted pathogens,^8 9^ increasing pressure for their routine use in asymptomatic populations.

Criteria for assessing the appropriateness of screening for a disease in the population include requirements that the disease is an important public health problem and that screening has been shown to do more good than harm.^10^ Precise estimates of the prevalence of *M. genitalium* in asymptomatic people in the general population are needed to assess public health importance and as input data for mathematical modelling studies that can investigate the potential effects of screening interventions on STI prevalence.^11^ The population prevalence of *M. genitalium* has not been ascertained systematically, to our knowledge. Non-systematic reviews have reported prevalence estimates ranging from 0.7% to 3.3% in the general population^1^ and from zero to 20% in a range of female study populations described as ‘low risk’.^12^ The frequency of *M. genitalium* infection is also of interest in specific populations whose behaviour places them at high risk of STI, such as men who have sex with men (MSM) and commercial sex workers (CSWs) and pregnant women, in whom transmission of infection to a fetus might have adverse consequences. The primary objective of this systematic review was to estimate the prevalence of *M. genitalium* in the general population. Secondary objectives were to estimate *M. genitalium* prevalence in specific groups: MSM, CSWs, pregnant women and consecutively enrolled attenders in clinics.

## Methods

We followed a predefined review protocol.^13^ This report presents the findings of the first of three review questions (prevalence of *M. genitalium*). Two other review questions (incidence and persistence of untreated *M. genitalium* infection) will be addressed in a separate report. We report the findings using the Preferred Reporting Items for Systematic Reviews and Meta-Analyses (PRISMA, research checklist online).^14^

### Eligibility criteria

We included studies that provided an estimate of the prevalence of *M. genitalium* infection in urogenital or rectal samples from women and men older than 13 years in any country from 1991 onwards, when the first NAAT was described.^1^ We included studies conducted among people in the general population or among attenders at healthcare settings that used NAAT to detect *M. genitalium*. Eligible study designs were cross-sectional studies and baseline data in cohort studies and randomised controlled trials, published as full papers, abstracts or conference posters. We excluded laboratory studies, studies restricted to people with a specific condition, for example, men with urethritis, women with abnormal cervical smears and women with pregnancy complications. Studies need to be large enough to estimate prevalence with sufficient precision.^15^ Studies with small sample sizes result in imprecise estimates that tend to be of lower methodological quality than large studies.^16^ We decided by consensus that we wanted to include at least 20 studies in the review. After assessing the sample sizes reported in the abstracts of identified records, we determined that inclusion of studies with 500 participants or more would result in at least 20 studies in the review.

### Information sources and search strategy

We searched Medline, Embase, African Index Medicus, IndMED and LILACS databases from 1 January 1991 to 12 July 2016 without language restrictions. The full search strategy for Medline and Embase is provided in  online supplementary text S1. The other databases were searched using only the term ‘*Mycoplasma genitalium*’. We used Endnote (V.7; Thomson Reuters) to import, de-duplicate and manage retrieved records.

10.1136/sextrans-2017-053384.supp1 Supplementary file 1

### Study selection

Two reviewers (LB, MC) independently screened the identified records using prepiloted checklists to assess eligibility, first of abstracts and titles and then of full text records. Differences were resolved by discussion or adjudication by a third reviewer (NL). When multiple records reported on the same study population, we defined a primary record to represent the study, based on a combination of the following factors: description as a main paper by the authors, most detailed report of methods, prevalence reported as the main result and date of publication.

### Data extraction

Two researchers extracted data independently (LB, DE-G, HA, G-RL, MC) for every included study, using a piloted extraction form in an online database (Research Electronic Data Capture, REDCap, Vanderbilt University, Tennessee). We resolved differences by discussion. The data extraction form included items about study design, demographic characteristics, sample size, methods of participant selection and specimen collection, response rates, number of infected participants and number tested and reported prevalence estimates (with 95% CIs) overall and for prespecified subgroups. If samples were taken from more than one anatomical site, we used the value for the site with the highest proportion of positive tests.

We also recorded a measure of the level of development of the country in which the study was done using the Human Development Index (HDI) 2015 dataset,^17^ which we categorised as higher (combining very high and high) or lower (medium and low). We defined studies a priori as ‘general population’ if they used any method to draw a random sample from the population of a whole country or a region, or as ‘community based’ if participants were enrolled outside healthcare settings but used non-random methods such as convenience sampling, snowball or respondent-driven sampling. Studies conducted in healthcare settings were coded according to their study population: clinic attenders, pregnant women, MSM and female CSWs. Studies that had enrolled participants from both healthcare settings and the community and did not stratify results were coded as clinic-based studies. We labelled studies according to the country in which the fieldwork was done and use these as study names in the text, tables and figures (online supplementary table S1). If there was more than one study from the same country, we assigned numbers after the country name. We generated separate strata within studies if they included participants from more than one country or from more than one relevant population subgroup, for example, MSM and heterosexual adults.

### Risk of bias in individual studies

To evaluate the individual studies, we adapted an instrument from another systematic review of studies of *Chlamydia trachomatis* prevalence (online supplementary text S2).^18^ Two reviewers independently assessed each item as being at high, low or uncertain risk of bias. Differences were resolved by discussion.

### Summary measure and synthesis of results

The outcome was the estimated prevalence (and 95% CI), defined as the number of specimens with a positive *M. genitalium* test result divided by the number of eligible participants with a valid test result. Where possible, we confirmed the published values using raw numbers reported in the publication. In studies that reported weighted prevalence estimates and CIs or where raw numbers were not available, we used the information reported by the authors. We calculated survey response rates, whenever possible, by dividing the number of participants tested by the number of eligible people asked to participate.

We initially examined the estimates of *M. genitalium* prevalence visually in forest plots. We stratified studies, based on a previous study showing factors that contribute to heterogeneity in estimates of *C. trachomatis* prevalence,^18^ by sampling method (random sample of the general population, community based or clinic based), study population (general population, pregnant women, MSM, CSW), HDI (higher or lower) and, where reported, by sex and age of participants as under 25 years or 25 years and older.

We used the I^2^ statistic to assess heterogeneity that was not due to random variation.^19^ Heterogeneity was considered moderate or high when I^2^ was greater than 50% or 75%, respectively. We used random effects meta-analysis to combine prevalence estimates where appropriate, assuming that, even when results were stratified, there might be real differences in *M. genitalium* prevalence between studies. We log-transformed the prevalence estimates and 95% CI before meta-analysis and back-transformed the summary average prevalence (and 95% CI) to the natural scale. We did not conduct meta-analysis on the logit scale because the log odds and CIs could not be obtained from studies that reported weighted prevalence estimates. We did a meta-regression analysis to examine possible factors (HDI, use of probability sampling, sample size, response rate, sex and use of adequate sample and target populations) contributing to heterogeneity in general population and clinic based studies. Analyses were done using the ‘metan’ and ‘metareg’ commands in Stata (Stata V.13; Stata, Austin, Texas, USA).

## Results

### Search results

We screened the titles and abstracts of 3316 unique records published after 1991 and the full text of 833 studies (online supplementary figure S1). A total of 63 records were included with participants who were sampled at random from the general population^20–25^ or using alternative community based methods,^26–30^ MSM and male-to-female transgendered,^31–36^ female CSWs^37–41^ and pregnant women.^42–45^ Of these, 37 studies included patients attending healthcare settings.^8 46–81^ We report results using the country name and number of the study or subgroup within a study. We did not include any studies conducted in male sex workers.

Table 1 shows that most characteristics of included studies were similar to those of studies excluded because the sample size was below 500 (details in online supplementary data). The distribution of included and excluded studies was broadly similar. Eight of the excluded studies included participants from the community, but all studies that used probability based sampling methods were included.

10.1136/sextrans-2017-053384.supp2 Supplementary file 2

**Table 1:** Characteristics of included and excluded studies

Characteristic	Included records		Excluded records	
	n=63*	(%)	n=113	(%)
Population				
General population	6	9.5	0	0.0
Community	5	7.9	8	7.1
Clinic based or mixed	37	58.7	65	57.5
Female commercial sex workers	5	7.9	11	9.7
Men who have sex with men	8	12.7	6	5.3
Pregnant women	4	6.3	9	8.0
Other	0	0.0	6	5.3
Unclear/not reported	0	0.0	8	7.1
Human Development Index of country				
Very high	44	69.8	65	57.5
High	6	9.5	25	22.1
Medium	7	11.1	7	6.2
Low	5	7.9	9	8.0
Multiple countries	1	1.6	2	1.8
Unclear	0	0.0	5	4.4
Sex		0.0		0.0
Women and men	25	39.7	18	15.9
Women only	23	36.5	61	54.0
Men only	15	23.8	33	29.2
Unclear	0	0.0	1	0.9
Sample size				
<500	0	0.0	113	100.0
500–1000	37	58.7	NA	NA
1001–2000	13	20.6	NA	NA
2001–3000	4	6.3	NA	NA
3001–4000	2	3.2	NA	NA
4001–5000	2	3.2	NA	NA
5001–10 000	4	6.3	NA	NA
>10 000	1	1.6	NA	NA
Publication year				
Before 2000	0	0.0	8	7.1
2001–2004	5	7.9	2	1.8
2005–2008	13	20.6	17	15.0
2009–2012	23	36.5	34	30.1
2013–2016	22	34.9	52	46.0
Percent positive tests				
Median %, (range)	8.1 (0.3–26.3)		8.0 (0.0–63.4)	

*Three records report on more than one study population: Netherlands 1 and Netherlands 2, Germany 2 and Germany 3, Honduras 3 and Guatemala 1.

NA, not applicable.

### Risk of bias in individual studies

No study was at low risk of bias in all domains (online supplementary figure S2). The studies at lowest risk of bias were those that used probability sampling in the general population. Only one study compared responders and non-responders and that study found differences between these groups.^25^ Reporting of complete results, including CIs and baseline data, was considered adequate in 22 studies.

### Studies in the general population and community

**Figure 1:**
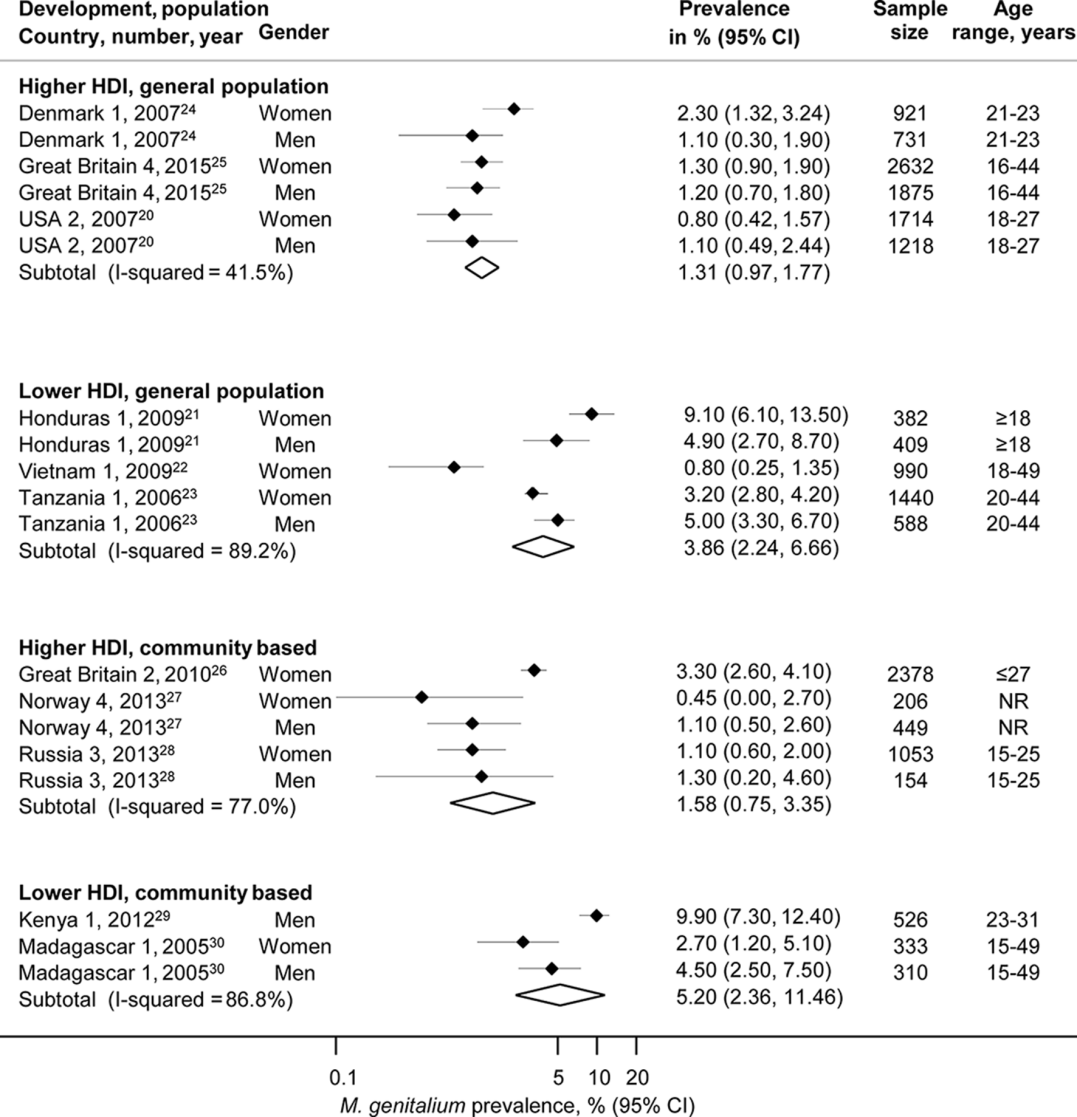
Estimated prevalence of *Mycoplasma genitalium* in randomly selected samples of the general population or in other community-based samples, by Human Development Index (HDI). Solid diamond and lines show the point estimate and 95% CIs for each study. The diamond shows the point estimate and 95% CIs of the summary estimate. The prevalence estimates are plotted on a logarithmic scale. NR, not reported.

We included 11 studies, 6 of which were in countries with higher HDI (Denmark 1,^24^ Great Britain 2 and Great Britain 4,^25 26^ Norway 4,^27^ Russian Federation 3^28^ and USA 2,^20^ n=13 331) and five in countries with a lower (Honduras 1,^21^ Vietnam 1,^22^ Kenya 1,^29^ Madagascar 1,^30^ Tanzania 1,^23^ n=4978) HDI (figure 1, online supplementary table S2).

The summary average general population prevalence of *M. genitalium* in three studies in countries with higher HDI was 1.3% (95% CI 1.0% to 1.8%, I^2^ 41.5%, n=9091, figure 1), with low between-study heterogeneity in three studies (one region in Denmark 1^24^ or the whole population in Great Britain 4 and USA 2).^20 25^ In three studies in higher HDI countries that enrolled participants using convenience sampling methods from subnational communities (n=4240, online supplementary table S2), between study heterogeneity was higher than in the studies that used random sampling methods, but the summary average prevalence was similar (1.6%, 95% 0.8% to 3.4%, I^2^ 77.0%).^26–28^ There were too few estimates from adults aged 25 years and over to compare *M. genitalium* prevalence between age groups. Among adults under 25 years, average *M. genitalium* prevalence was 1.7% (95% CI 1.0% to 2.6%, I^2^ 80.3%) in women and 0.3% in men (0.1% to 1.4%, I^2^ 91.3%) (online supplementary figure S3).

The surveys from five countries with lower HDI enrolled very different populations and *M. genitalium* prevalence estimates were more variable (figure 1, online supplementary table S2).^21–23 29 30^ The summary estimate of prevalence in three studies that used probability sampling was 3.9% (2.2 to 6.7, I^2^ 89.2%) and, in two studies that used other methods to enrol participants from community settings, 5.2% (2.4 to 11.5, I^2^ 86.8%).

In a meta-regression analysis that compared characteristics of all studies in adults in the general population, there was some statistical evidence to suggest higher estimates of *M. genitalium* prevalence in countries with lower than higher HDI (difference 3.1%, 95% CI −0.1% to 6.3%, P=0.057) but no statistical evidence of a difference by sex (0.9%, 95% CI −1.6% to 3.3% P=0.47) or for other study related variables that were examined (online supplementary table S3).

### Pregnant women in antenatal clinics and women in the general population

**Figure 2:**
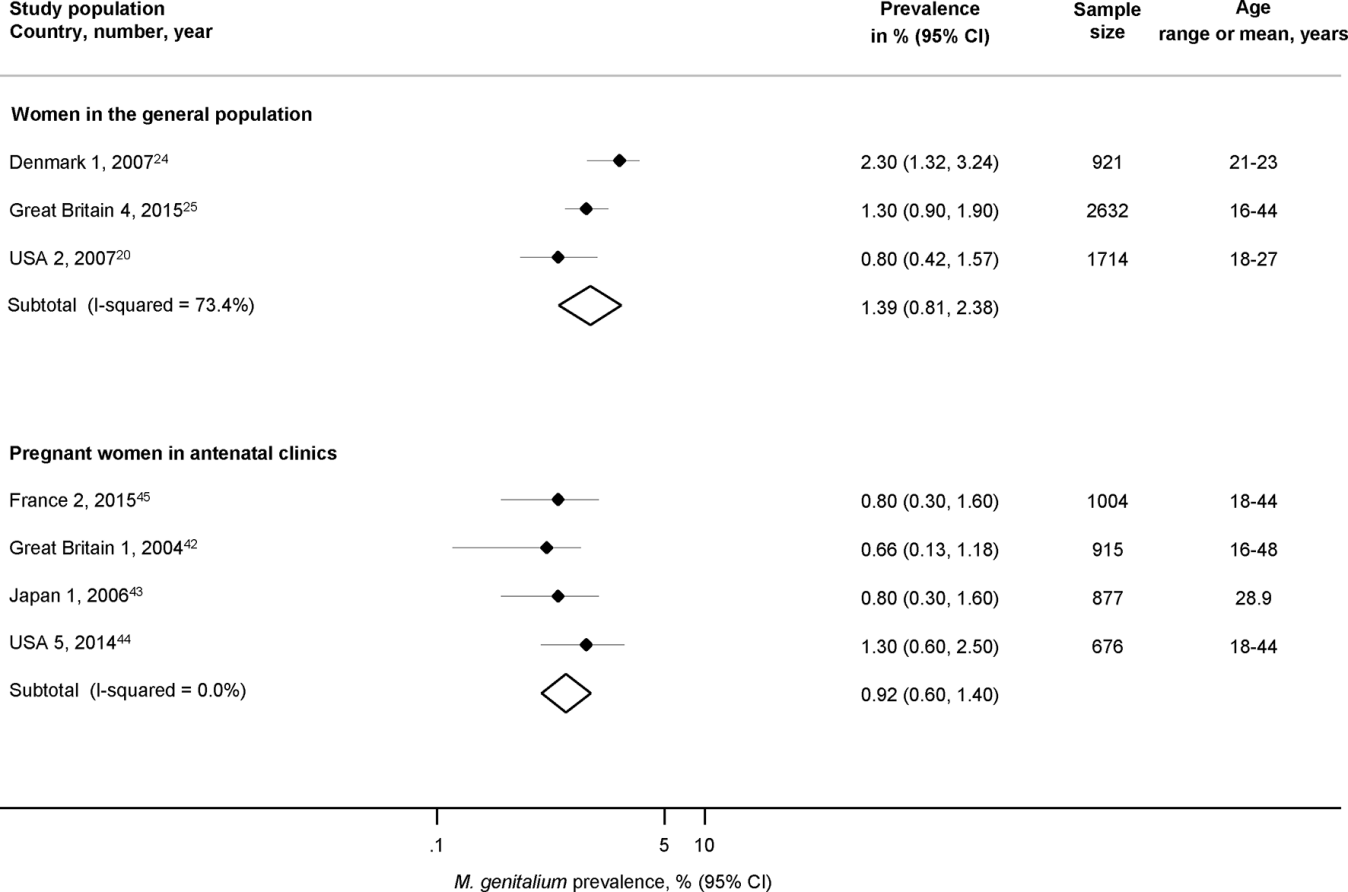
Estimated prevalence of *Mycoplasma genitalium* in pregnant women in antenatal clinics and in randomly selected samples of women in the general population. Solid diamond and lines show the point estimate and 95% CIs for each study. The diamond shows the point estimate and 95% CIs of the summary estimate. The prevalence estimates are plotted on a logarithmic scale.

We included four studies in pregnant women before 14 weeks’ gestation, all in countries with higher HDI (n=3472, age range 16 to 48 years; France 2,^45^ Great Britain 1,^42^ Japan 1^43^ and USA 5^44^; figure 2, online supplementary table S3) (0.9%, 95% CI 0.6% to 1.4%, I^2^ 0%). The estimated prevalence was slightly lower than in the three studies in women in the general population (1.4%, 95% CI 0.8% to 2.4%, I^2^ 73.4%) but CI overlapped.

### MSM and female CSW in community based and clinic based studies

**Figure 3:**
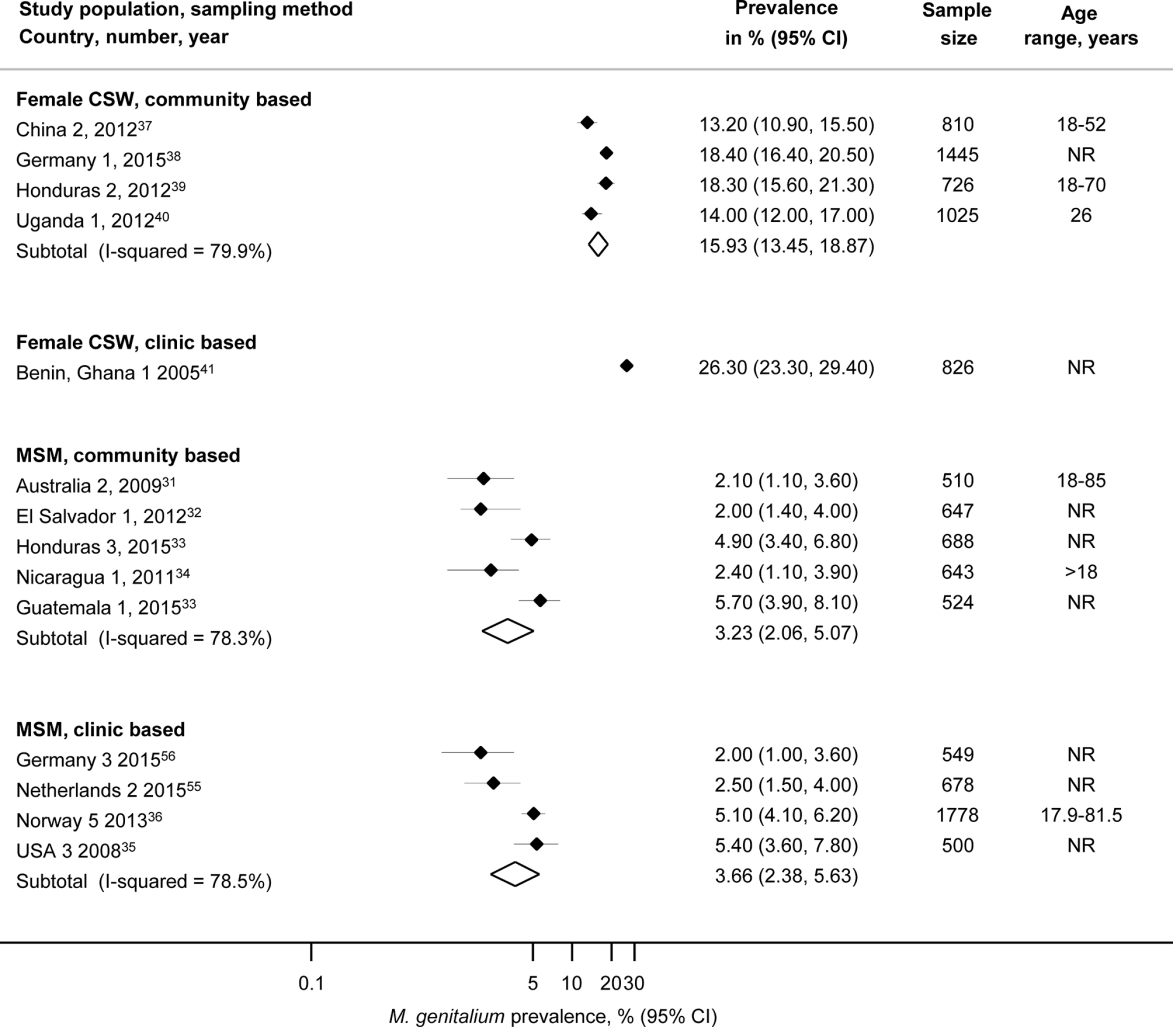
Estimated prevalence of *Mycoplasma genitalium* in community based and clinic based samples of men who have sex with men and female sex workers. CSW, commercial sex worker; MSM, men who have sex with men; NR, not reported. Solid diamond and lines show the point estimate and 95% CIs for each study. The diamond shows the point estimate and 95% CIs of the summary estimate. The prevalence estimates are plotted on a logarithmic scale.

Five studies from four records enrolled MSM from the community (figure 3, online supplementary table S4) in specific areas in Australia 2,^31^ El Salvador 1,^32^ Guatemala 1 and Honduras 3,^33^ and Nicaragua 1,^34^ (n=3012). The summary average prevalence in these studies was 3.2% (95% CI 2.1% to 5.1%, I^2^ 78.3%) with moderate between study heterogeneity. The summary average estimate of *M. genitalium* prevalence in MSM enrolled from clinics in Germany 3,^56^ the Netherlands 2,^55^ Norway 5^36^ and USA 3^35^ was 3.7% (95% CI 2.4% to 5.6%, I^2^ 78.5%).

Four studies enrolled female CSW in the community based studies in specific areas in southwest China 2,^37^ northern Germany 1,^38^ Honduras 2^39^ and Uganda 1.^40^ Estimated *M. genitalium* prevalence was 15.9% (95% CI 13.5% to 18.9%, I^2^ 79.9%, n=4006), which was lower than in one study that enrolled women from a clinic in Benin and Ghana 1.^41^

### Clinic based studies

We included 37 studies from 14 countries, of which 24 were from Australia, Great Britain, Norway, South Korea and Sweden (online supplementary table S5 and figure S4). Estimates of *M. genitalium* were very heterogeneous (I^2^>95%), except for in three studies that only included patients without symptoms^8 51 65^ (0.8%, 95% CI 0.4% to 1.4%, I^2^ 0%, n=2889). Most study populations included patients both with and without symptoms. Point estimates varied widely, both in studies that enrolled patients consecutively (range 1.0%^52^ to 8.7%,^64^ I^2^ 96.1%) and studies in which the enrolment procedure was not clearly described (range 0.6%^79^ to 12.6%,^62^ I^2^ 98.2%), and we did not combine results. There was no consistent difference in prevalence estimates from specialist STI clinics and general practice or primary healthcare clinics (online supplementary figure S4 and table S5).

## Discussion

### Main findings

In large nationally representative surveys conducted in very highly developed countries, the summary average prevalence estimate of *M. genitalium* was 1.3% (95% CI 1.0% to 1.8%, three studies, I^2^ 41.5%) in adults aged 16 to 44 years with no statistical evidence of a difference between men and women (P=0.47). Summary prevalence estimates were, in the following specific subpopulations: pregnant women 0.9% (0.6 to 1.4%), MSM in community samples 3.2% (2.1 to 5.1%, five studies, I^2^ 78.3%) and MSM in clinic based samples 3.7% (2.4 to 5.6%, four studies, I^2^ 78.5%). Prevalence estimates were higher in FSW, ranging from 13.2% in one community based study to 26.3% in one clinic based study. In clinic based surveys, prevalence estimates varied widely from 0.6% to 12.6% and were not combined.

### Strengths and limitations

The broad search strategy is a strength of this review. It allowed for identification of a wide range of different studies, and it is unlikely that we missed large studies. The a priori defined inclusion criteria allowed a clear selection process for the detected studies and duplicate screening and data extraction prevented data entry errors. By including only studies with 500 participants or more, we aimed to reduce the influence of small study biases that can distort results. This strategy included all studies that used methods to select random samples of the general population and provided summary estimates with little heterogeneity for general population samples in very highly developed countries, pregnant women and asymptomatic people attending outpatient healthcare settings. Although we explored between-study heterogeneity using meta-regression analysis, we did not identify factors that could explain a substantial proportion of the heterogeneity. Finally, we could not assess an earlier finding, in surveys of chlamydia prevalence,^18^ that lower response rates are associated with higher prevalence estimate because very few studies reported these results. Among studies that reported response rates, we did not find an association with *M. genitalium* prevalence (online supplementary table S3).

### Interpretation and comparison with other studies

To our knowledge, this is the first systematic review assessing the prevalence of *M. genitalium* in different population groups, including those outside healthcare settings. Our findings suggest that *M. genitalium* might be less prevalent than *C. trachomatis* in the general population, but comparison is not straightforward. In a systematic review of population based surveys of *C. trachomatis*, estimated prevalence in adults <27 years in high-income countries was 4.3% (95% CI 3.6% to 5.0%, I^2^ 0%) in women and 3.6% (95% CI 2.8% to 4.4%, I^2^ 6.2%) in men,^18^ compared with our summary estimates of less than 2% for *M. genitalium* in women and men <25 years old. Within studies that tested for both pathogens, prevalence estimates for *M. genitalium* and *C. trachomatis* were similar in Great Britain,^25^ but higher for *C. trachomatis* than *M. genitalium* in Denmark^24^ and the USA.^20^ It is, however, possible that *M. genitalium* prevalence has been underestimated because the sensitivity of NAATs is lower than previously believed.^9^ In general, age differences seem less marked among women for *M. genitalium* than for *C. trachomatis*, where prevalence after age 25 years is much lower than in younger women. Age specific patterns of *M. genitalium* were, however, difficult to discern with certainty, largely because population-based studies that provided age-stratified estimates used non-comparable age groups and only two had estimates for participants older than 25 years.^25 27^

In clinic based surveys, participant selection methods and characteristics differed substantially between different types of clinics and countries. *M. genitalium* prevalence estimates were consistent and comparable (or even lower) than in general population based surveys in studies that only enrolled asymptomatic patients or pregnant women in antenatal clinics. Among MSM, estimated *M. genitalium* prevalence was similar in community based and clinic based studies.

### Implications for clinical practice, policy and research

This systematic review provides evidence about the prevalence of *M. genitalium* that can be used in mathematical modelling studies to investigate the potential impact of screening interventions^82^ and to inform testing guidelines for infection.^83^ The trend for molecular diagnostic tests to include targets that identify multiple sexually transmitted pathogens means that testing for asymptomatic *M. genitalium* infection will become more widespread. High levels of antimicrobial resistance in *M. genitalium* are already a concern,^84^ so estimates of prevalence are also needed for monitoring purposes if drug resistance promotes further spread of infections. The absence of randomised controlled trials that demonstrate a clinical benefit of screening and the increasing prevalence of resistance to azithromycin are reasons for restricting widespread testing for *M. genitalium*.^85^ The low estimated prevalence of *M. genitalium* in the general population, in pregnant women and in asymptomatic attenders in healthcare settings and absence of a clearly defined age group at higher risk of infection do not provide strong support for the appropriateness of universal or age based screening programmes for *M. genitalium* in these population groups.

**Key messages**

- Routine screening for *Mycoplasma genitalium* infection has been proposed, but prevalence rates are not well established.
- In samples from the general population, the summary prevalence estimate is 1.3% in countries with higher development and 3.9% in countries with lower development.
- *M. genitalium* prevalence in the general population and differences in prevalence by age appear to be less than for *Chlamydia trachomatis.*
- The low prevalence estimates in the general population, pregnant women and asymptomatic clinic based patients do not support universal screening for *M. genitalium.*
