# Supplementary material for: Prevalence of Mycoplasma genitalium in different population groups: systematic review andmeta-analysis
Source: Sex Transm Infect. 2018 Feb 9;94(4):255–62. doi: 10.1136/sextrans-2017-053384 (PMC5969327; doi:10.1136/sextrans-2017-053384)

Supplementary material

The prevalence of *Mycoplasma genitalium* in different population groups: systematic review and meta-analysis

Lukas Baumann,[1] Manuel Cina,[1] Dianne Egli-Gany,[1] Myrofora Goutaki,[1] Florian Halbeisen,[1] Gian-Reto Lohrer,[1] Hammad Ali,[2] Pippa Scott,[1,3] Nicola Low[1]

1. Institute of Social and Preventive Medicine, University of Bern, Bern, Switzerland;
2. Kirby Institute, University of New South Wales, Sydney, Australia;
3. Department of Pathology, University of Otago, Christchurch, New Zealand

Contents

Supplementary text, Text S1 and Text S2 2

Text S1. Medline search strategy 2

Text S2. Risk of bias assessment 3

Supplementary tables, Table S1 to Table S5 4

Table S1. Included studies, by reference number and study name 4

Table S2. Characteristics of studies of *M. genitalium* prevalence in general population and Community based samples 6

Table S3. Meta-regression model of potential sources of heterogeneity in studies of *M. genitalium* prevalence 8

Table S4. Characteristics of studies of *M. genitalium* prevalence in pregnant women, men who have sex with men and female sex workers, by study setting 10

Table S5. Characteristics of studies of *M. genitalium* prevalence in healthcare clinic based settings, by symptom status of patients 12

Supplementary figures, Figure S1 to Figure S4 15

Figure S1. Flow chart of included and excluded studies 15

Figure S2. Risk of bias assessment of included studies 16

Figure S3. Forest plot of studies of *M. genitalium* prevalence, by age and sex in general population samples 18

Figure S4. Forest plot of studies of *M. genitalium* prevalence in healthcare clinic based settings, by symptom status of patients and enrolment process 19

Supplementary material

Supplementary text, Text S1 and Text S2

Text S1. Medline search strategy

1: „Mycoplasma genitalium"[Mesh]
2: Mycoplasma genitalium
3: 1 OR 2
4: "Mycoplasma Infections"[Mesh]
5: Mycoplasma
6: Mycoplasm*
7: 4 OR 5 OR 6
8: "Reproductive Tract Infections"[Mesh]
9: genital tract
10: reproductive tract
11: "Salpingitis"[Mesh]
12: Salpingitis
13: "Endometritis"[Mesh]
14: Endometritis
15: "Parametritis"[Mesh]
16: Parametritis
17: "Oophoritis"[Mesh]
18: Oophoritis
19: Ovary
20: Metritis
21: Pelviperitonitis
22: "Pelvic Inflammatory Disease"[Mesh]
23: p.i.d.
24: pelvis
25: pelvic
26: Adnexitis
27: "Sexually Transmitted Diseases"[Mesh]
28: sexually transmitted
29: STD
30: STDs
31: VD
32: Sexual disease transmission
33: Veneral
34: Venereal
35: Genital*
36: Vagina*
37: Endometri*
38: Cervix
39: Cervical*
40: Urethra*
41: Fallopian
42: tuba*
43: tube
44: tubes
45: 8 OR 9 OR 10 OR 11 OR 12 OR 13 OR 14 OR 15 OR 16 OR 17 OR 18 OR 19 OR 20 OR 21
OR 22 OR 23 OR 24 OR 25 OR 26 OR 27 OR 28 OR 29 OR 30 OR 31 OR 32 OR 33 OR 34 OR 35 OR 36 OR 37 OR 38 OR 39 OR 40 OR 41 OR 42 OR 43 OR 44
46: 3 OR (7 AND 45)

Filters: 1981/01/01 – Now Humans

Supplementary material

Text S2. Risk of bias and reporting assessment

Target population clearly defined?
- Yes
- No
- Unclear

Source population clearly defined?
- Yes
- No
- Unclear

Source population adequate sample of target population?
- Yes
- No
- Unclear

Similar socio-demographic attributes of responders and non-responders:
- responders compared and similar and non-responders attributes
- responders and non-responders compared and not similar attributes
- responders and non-responders not compared

Was a probability sampling used?
- Yes
- No
- Unclear

Was a sample size calculation reported?
- Adequate
- Inadequate
- Not reported

Was the achieved sample size at least as good as in the sample size calculation?
- Adequate
- Inadequate
- Unclear

Can we be confident in the assessment of exposure?
- Yes
- No

Data provided to calculate prevalence?
- Yes
- No
- Weighted prevalence estimate

Confidence intervals included for prevalence?
- Yes
- No

Data provided on both number of people asked to participate and number of people participating?
- Yes
- No

Response rate:
- over 80%
- 70-80%
- below 70%
- unclear
- not reported

Supplementary material

Supplementary tables, Table S1 to Table S5

Table S1. Included studies, by reference number and study name

Ref no.	Study country, number	First author	Publication year	Sample	Population
8	South Korea 1	Kim SJ	2011	Clinic	Women and men
20	USA 2	Manhart LE	2007	General population	Women and men
21	Honduras 1	Paz-Bailey G	2009	General population	Women and men
22	Vietnam 1	Olsen B	2009	General population	Women
23	Tanzania 1	Kapiga SH	2006	General population	Women and men
24	Denmark 1	Andersen B	2007	General population	Women and men
25	Great Britain 4	Sonnenberg P	2015	General population	Women and men
26	Great Britain 2	Oakeshott P	2010	Community based	Women
27	Norway 4	Jensen AJ	2013	Community based	Women and men
28	Russia 3	Shipitsyna E	2013	Community based	Women and men
29	Kenya 1	Mehta SD	2012	Community based	Men
30	Madagascar 1	Leutscher PDC	2005	Community based	Women and men
31	Australia 2	Bradshaw CS	2009	Community based	MSM
32	El Salvador 1	Creswell J	2012	Community based	MSM
33	Guatemala 1	Ham D	2015	Community based	MSM
33	Honduras 3		2015	Community based	MSM
34	Nicaragua 1	Hernandez F	2011	Community based	MSM
35	USA 3	Francis SC	2008	Clinic	MSM
36	Norway 5	Reinton N	2013	Clinic	MSM
37	China 2	Xiang Z	2012	Community based	Female CSW
38	Germany 1	Jansen K	2015	Community based	Female CSW
39	Honduras 2	Johnston LG	2012	Community based	Female CSW
40	Uganda 1	Vandepitte J	2012	Community based	Female CSW
41	Benin, Ghana 1	Pepin J	2005	Clinic	Female CSW
42	Great Britain 1	Oakeshott P	2004	Clinic	Pregnant women
43	Japan 1	Kataoka S	2006	Clinic	Pregnant women
44	USA 5	Agger WA	2014	Clinic	Pregnant women
45	France 2	Peuchant O	2015	Clinic	Pregnant women
46	Australia 1	McKechnie ML	2009	Clinic	Women and men
47	Australia 3	Walker J	2011	Clinic	Women
48	Australia 4	Lusk MJ	2011	Clinic	Women
49	China 1	Bao T	2010	Clinic	Men

50	France 1	Sednaoui P	2011	Clinic	Women and men
51	France 3	Clarivet B	2014	Clinic	Women and men
52	Great Britain 3	Jalal H	2013	Clinic	Women and men
53	Great Britain 5	Svenstrup HF	2014	Clinic	Women
54	Great Britain 7	Slack R	2014	Clinic	Men
55	Netherlands 1	Van der Veer C	2015	Clinic	Heterosexual men
55	Netherlands 2		2015	Clinic	MSM
56	Germany 2	Lallemand A	2015	Clinic	Heterosexual women and men
56	Germany 3			Clinic	MSM
57	Norway 1	Moi H	2009	Clinic	Men
58	Norway 2	Moi H	2009	Clinic	Women
59	Norway 3	Nilsen E	2011	Clinic	Women and men
60	Norway 6	Hartgill U	2015	Clinic	Women
61	Norway 7	Reinton N	2015	Clinic	Women and men
62	Russia 1	Khryanin A	2011	Clinic	Women and men
63	Russia 2	Berle LM	2012	Clinic	Women and men
64	South Africa 1	Hay B	2015	Clinic	Women
65	South Korea 2	Choi JY	2013	Clinic	Men
66	South Korea 3	Kim Y	2014	Clinic	Women
67	Sweden 1	Falk L	2003	Clinic	Women and men
68	Sweden 2	Falk L	2004	Clinic	Men
69	Sweden 3	Jensen JS	2004	Clinic	Women and men
70	Sweden 4	Mellenius H	2005	Clinic	Women and men
71	Sweden 5	Anagrius C	2005	Clinic	Women and men
72	Sweden 6	Jurstrand M	2005	Clinic	Women and men
73	Sweden 7	Hogdahl M	2007	Clinic	Women and men
74	Sweden 8	Edberg A	2008	Clinic	Women and men
75	Sweden 9	Bjartling C	2012	Clinic	Women
76	Uganda 2	Tobian AA	2014	Clinic	Women
77	USA 1	Manhart LE	2003	Clinic	Women
78	USA 4	Hancock EB	2010	Clinic	Women
79	Venezuela 1 2013	Peralta-Arias RD	2013	Clinic	Women
80	Canada 1	Gesink D	2016	Clinic	Women, men, transgender
81	Great Britain 6	Leung A	2006	Clinic	Men

Abbreviations: CSW, commercial sex worker; MSM, men who have sex with men

Supplementary material

Table S2. Characteristics of studies of *M. genitalium* prevalence in general population and Community based samples

Country, number	Year	First author	Study type	Specimen type	Study setting and population	Sample size	Gender	Age, years range/median/mean
**Very high or high development index**	**General Population**							
Denmark 1	2007	Andersen B	Cross-sectional	M: Urine<br>W: Vagina	Random sample of men and women who participated in a population- based screening programme on Chlamydia	1652	M&W	21-23/NR/NR
Great Britain 4	2015	Sonnenberg P	Cross-sectional	Urine	A stratified, clustered sample of sexually experienced people living in the UK aged 16 - 44, with postcode sectors as the primary sampling units	4507	M&W	16-44/NR/NR
USA 2	2007	Manhart LE	Cross-sectional	Urine	Randomly sampled in house respondents of the Wave III of the national Add Health study, recruited from high schools and junior high schools	2932	M&W	18-27/NR/NR
**Middle or low development index**	**General population**							
Honduras 1	2009	Paz-Bailey G	Cross-sectional	M: Urine<br>W: Vagina	Multistage cluster sampling from the eight largest Garifuna (ethnic minority) communities in the three departments with the highest concentration of Garifunas in Honduras	791	M&W	>18/30/NR
Tanzania 1	2006	Kapiga SH	Cross-sectional	Urine	Stratified random sample of women aged 20-44 years and their husbands/male regular partners selected from different clusters within Moshi urban district, Tanzania	2028	M&W	20-44/NR/NR
Vietnam 1	2009	Olsen B	Cross-sectional	Cervix	Stratified random sample of married women in a rural geographical surveillance site in Vietnam	990	W	18-49/NR/NR
**Very high or high development index**	**Community setting**							
Great Britain 2	2010	Oakeshott P	Baseline cohort	Vagina	Convenient sample of female students from 20 London universities and colleges	2378	W	NR/NR/20
Norway 4	2013	Jensen AJ	Cross-sectional	Urine	Students recruited from three colleges in three northernmost counties in Norway	655	M&W	NR/NR/NR

Supplementary material

Country, number	Year	First author	Study type	Specimen type	Study setting and population	Sample size	Gender	Age, years range/median/mean
Russia 3	2013	Shipitsyna E	Cross-sectional	M: Urine<br>W: Vagina	Consecutive sample of sexually active attendees of a youth centre in St. Petersburg, Russia	1207	M&W	15-25/NR/20
**Middle or low development index**	**Community setting**							
Kenya 1	2012	Mehta SD	Cross-sectional	Urine	Sample of young men drawn from a RCT on male circumcision, recruited via local newspapers, radio, fliers, and street shows by drama and musical groups. Enrolled participants recruited further participants for screening	526	M	23-31/NR/NR
Madagascar 1	2005	Leutscher PDC	Baseline RCT	Urine	Sample of adults aged 15-49 years in rural villages (Ambodikatakata, Ambodimanga, Ankatoko, and Tanambao) on Madagascar's northwest coast	643	M&W	NR/29/NR

Abbreviations: M, men; NR, not reported; W, women

Supplementary material

Table S3. Meta-regression model of potential sources of heterogeneity in studies of *M. genitalium* prevalence

**Population based studies**									
**Meta-Regression**						**Meta-analysis (random effects)**			
**Variable**	**Category-description**	**Coefficient**	**95% CI**	**p-value**	**Residual I^2**	**Number of studies**	**Summary estimate, %**	**95% CI**	**I^2**
Human development index	Medium + low	3.1	-0.1, 6.3	0.057	92.65%	5	4.8	2.3, 7.3	95.6%
	cons (Very high + high)	1.6	-0.5, 3.7	0.125		6	1.5	0.9, 2.2	83.9%
Was a probability sample used?	Yes	-1.1	-5.0, 2.9	0.549	92.73%	6	2.1	1.2, 3.0	91.5%
	cons (No + unclear)	3.6	0.6, 6.5	0.023		5	3.4	1.6, 5.2	93.8%
Did they report the result with CIs?	Yes	1.5	-2.8, 5.9	0.454	93.16%	8	3.0	1.9, 4.0	94.2%
	cons (No)	1.9	-1.8, 5.6	0.283		3	1.7	0.5, 2.9	79.8%
Is the source population an adequate sample of the target population?	Yes	-2.5	-6.6, 1.6	0.637	91.61%	8	1.9	1.2, 2.7	89.1%
	cons (No + unclear)	4.8	1.3, 8.3	0.013		3	4.8	2.0, 7.5	95.4%
Response rate ≥80	Response rate ≥80	4.5	-2.1, 11.2	0.157	93.22%	1	7.1	4.7, 9.5	N/A
	cons (< 80 or unclear)	2.6	0.7, 4.4	0.011		10	2.3	1.5, 3.1	92.0%
Sample size ≥1000	Sample size ≥1000	-2.1	-5.8, 1.6	0.229	93.21%	6	2.0	1.2, 2.8	90.8%
	cons (<1000)	6.3	0.2, 12.4	0.045		5	4.1	1.8, 6.5	94.9%
Sex	Men	0.9	-1.6, 3.3	0.470	88.81%	9	3.0	1.7, 4.2	88.8%
	cons (Women)	2.2	0.6, 3.9	0.012		10	2.0	1.2, 2.7	88.8%
**Clinic based studies**									
**Meta-Regression**						**Meta-analysis (random effects)**			
**Variable**	**Category-description**	**Coefficient**	**95% CI**	**p-value**	**Residual I^2**	**Number of studies**	**Pooled prevalence, %**	**95% CI**	**I^2**
Human development index	Medium + low	1.8	-2.5, 6.1	0.398	98.02%	2	6.0	0.8, 11.2	93.9%
	cons (Very high + high)	4.1	3.1, 5.1	0.000		32	4.1	3.3, 4.8	98.0%
Was a probability sample used?	Yes	2.9	-2.9, 8.6	0.315	97.89%	1	7.0	5.2, 9.1	N/A
	cons (No + unclear)	4.1	3.2, 5.0	0.000		36	4.1	3.4, 4.8	97.9%

Did they report the result with CIs?	Yes	-0.7	-3.0, 1.6	0.555	97.82%	7	3.5	2.2, 4.9	95.5%
	cons (No)	4.3	3.3, 5.3	0.000		30	4.3	3.5, 5.2	98.0%
Is the source population an adequate sample of the target population?	Yes	0.6	-1.4, 2.7	0.523	97.85%	10	4.7	2.4, 7.0	99.0%
	cons (No + unclear)	4.0	3.0, 5.1	0.000		27	3.9	3.3, 4.6	96.1%
Response rate ≥80	Response rate >=80	-0.4	-3.1, 2.2	0.501	97.75%	5	3.7	2.0, 5.4	96.1%
	cons (< 80 or unclear)	4.3	3.3, 5.2	0.376		32	4.2	3.4, 5.0	97.9%
Sample size ≥1000	Sample size ≥1000	-0.1	-1.9, 1.7	0.893	97.90%	17	4.1	3.1, 5.2	98.8%
	cons (<1000)	4.4	1.5, 7.2	0.004		20	4.2	3.2, 5.3	93.2%
Sex	Men	0.0	-1.3, 1.3	0.977	93.78%	23	3.9	3.2, 4.6	91.6%
	cons (Women)	4.0	3.1, 4.9	0.000		27	3.8	3.2, 4.5	94.9%

Abbreviations: CI, confidence intervals; cons, constant

Note: cons is the reference group, with the columns ‘Coefficient’ and ‘95% CI’ showing the average summary estimate of prevalence and 95% CI. The value for the other (non-reference) category is the difference in average prevalence between the groups; N/A, no I^2 available for these variables because only one study was included in group

Supplementary material

Table S4. Characteristics of studies of *M. genitalium* prevalence in pregnant women, men who have sex with men and female sex workers, by study setting

Country, number	Year	First author	Study type	Specimen type	Study setting and population	Sample size	Gender	Age, years range/median/mean
**MSM, community based**								
Australia 2	2009	Bradshaw CS	Cross-sectional	Urine, rectum, throat	MSM attending six "sex on premises venues" in Melbourne	510	Men	18-85/39/NR
El Salvador 1	2012	Creswell J	Cross-sectional	Urine	MSM in San Salvador and San Miguel	647	Men	NR/NR/NR
Guatemala 1	2015	Ham D	Cross-sectional	Urine	MSM in Guatemala	524	Men	NR/NR/NR
Honduras 3	2015	Ham D	Cross-sectional	Urine	MSM in Honduras	688	Men	NR/NR/NR
Nicaragua 1	2011	Hernandez F	Cross-sectional	Urine	MSM in Nicaragua	643	Men	>18/NR/NR
**MSM, clinic based**								
Germany 3	2015	Lallemand A	Cross-sectional	Urine	MSM seeking HIV testing at local public health authorities in North Rhein-Westphalia	549	Men	NR/NR/NR
Netherlands 2	2015	Van der Veer C	Cross-sectional	Urine	MSM attending a sexual health clinic in Amsterdam	678	Men	NR/NR/41
Norway 5	2013	Reinton N	Cross-sectional	Urine, rectum	MSM attending two sexual health clinics in Oslo	1778	Men	18-82/35/NR
USA 3	2008	Francis SC	Cross-sectional	Rectum	MSM attending a sexual health clinic in San Francisco	500	Men	NR/NR/NR
**FSW, community based**								
China 2	2012	Xiang Z	Cross-sectional	Cervix	FSW from various sex-work venues	810	Women	18-52/NR/27
Germany 1	2015	Jansen K	Cross-sectional	Vagina	FSW from 292 different places of work	1445	Women	NR/NR/NR
Honduras 2	2012	Johnston LG	Cross-sectional	Vagina	FSW in four Honduran cities	726	Women	NR/NR/NR
Uganda 1	2012	Vandepitte J	Baseline cohort	Cervix	FSW from red-light areas in southern Kampala	1025	Women	NR/26/26
**FSW, clinic based**								
Benin, Ghana 1	2005	Pepin J	Cross-sectional	Cervix	FSW from sexual health clinics in Acca (Ghana), Cotonou and Port Novo (Benin)	826	Women	NR/NR/NR

**Pregnant women, antenatal clinics**								
France 2	2015	Peuchant O	Cross-sectional	Vagina	Pregnant women attending the Bordeaux University Hospital	1004	Women	18-44/30/NR
Great Britain 1	2004	Oakeshott P	Cross-sectional	Urine	Pregnant women (<10 weeks gestation) from 32 general practices and 5 family planning clinics	915	Women	16-48/NR/31
Japan 1	2006	Kataoka S	Baseline cohort	Vagina	Pregnant women with singleton pregnancies attending a university hospital	877	Women	NR/NR/29
USA 5	2014	Agger WA	Baseline cohort	Urine	Pregnant women attending 4 sites for initial antenatal visits in Milwaukee	676	Women	18-44/NR/NR

Abbreviations: FSW, female sex workers; MSM, men who have sex with men; NR, not reported

Supplementary material

Table S5. Characteristics of studies of *M. genitalium* prevalence in healthcare clinic based settings, by symptom status of patients

Country, number	Year	First author	Study type	Specimen type	Study setting and population	Sample size	Gender	Age, years range/median/mean	Number of symptomatic patients
**Asymptomatic only**									
France 3	2014	Clarivet B	Cross-sectional	Urine	Asymptomatic patients attending anonymous STI clinics	1381	W&M	<30/22/NR	0/1381 (0%)
South Korea 1	2011	Kim SJ	Cross-sectional	M: Urine W: Cervix	Sexually active asymptomatic patients attending hospital for general check-up	709	W&M	20-60/NR/45	0/709 (0%)
South Korea 3	2014	Kim Y	Cross-sectional	Cervix	Healthy Korean women visiting a hospital for general medical check-up	799	W	25-81/49/50	0/799 (0%)
**Consecutive patients, with and without symptoms**									
Australia 3	2011	Walker J	Baseline cohort	Vagina	Consecutive patients attending primary health care clinics in Australia	1116	W	16-25/21/NR	249/1116 (22%)
Australia 4	2011	Lusk MJ	Cross-sectional	Cervix	Consecutive patients from two STI clinics in Sydney	527	W	NR/NR/NR	NR
Canada 1	2016	Gesink D	Baseline cohort	Urine	Consecutive STI clinic attendees in Toronto, Ontario	1193	W&M &T	19-57/NR/33	M: T 442/884 (50%) W: 124/309 (40%)
Great Britain 3	2013	Jalal H	Cross-sectional	W: Cervix and urethra M: Urethra	Consecutive patients attending a GUM clinic in Cambridge	1718	W&M	12-87/W24,M27/NR	NR
Norway 6	2015	Hartgill U	Cross-sectional	Cervix	Consecutive patients attending an STI clinic for an STI screen	1097	W	NR/NR/NR	NR
Norway 7	2015	Reinton N	Cross-sectional	Unclear	Consecutive samples for CT screening from 3 STI clinics and 613 primary care clinics around Oslo	78505	W&M	13-79/NR/NR	NR
South Africa 1	2015	Hay B	Cross-sectional	Vagina, rectum, pharynx if oral sex	Consecutive female attendees of 25 selected Primary Health Care facilities	601	W	18-49/30/ NR	NR
Sweden 2	2004	Falk L	Cross-sectional	Urine	Consecutive patients attending Örebro University Hospital STI clinic	512	M	16-67/27/ NR	23/512 (45%)
Sweden 3	2004	Jensen JS	Cross-sectional	M: Urethra, urine W: Cervix, urethra, urine	Consecutive patients attending the outpatient STI clinic at Huddinge University Hospital	2605	W&M	NR/NR/NR	NR
Sweden 5	2005	Anagrius C	Cross-sectional	M: Urethra W: Urethra, cervix	Consecutive patients attending an STI clinic	946	W&M	14-67/NR/NR	W: 130/446 (29%) M: 125/501 (25%)
Sweden 6	2005	Jurstrand M	Cross-sectional	M: Urine W: Cervix	Consecutive attendees to the outpatient STI clinic at Örebro Hospital Sweden	699	W&M	15-58/W23,M27/W26,M28	NR
Sweden 7	2007	Hogdahl M	Cross-sectional	Urine	Consecutive patients attending STI clinics	833	W&M	17-52/NR/W22,M26	W: 112/405 (28%) M: 89/391 (23%)

Supplementary material

Country, number	Year	First author	Study type	Specimen type	Study setting and population	Sample size	Gender	Age, years range/median/mean	Number of symptomatic patients
Sweden 8	2008	Edberg A	Cross-sectional	M: Urine &/or urethra. W: Cervix &/or urine	Consecutive patients attending an STI clinic at the Central Hospital Karlstad, Sweden	679	W&M	17-82/W25,M27/NR	NR
Sweden 9	2012	Bjartling C	Cross-sectional	2003-2004: Urine & cervix, 2005-2008: Urine & vagina	Consecutive patients at emergency gynecological outpatient service, Skane University Hospital, Malmö	5519	W	15-46/NR/NR	NR
**Patient enrolment not clearly described**									
Australia 1	2009	McKechnie ML	Cross-sectional	Urine	Men with and without urethral symptoms attending two sexual health clinics in Sydney	529	M	19-76/35/37	277/529 (52%)
China 1	2010	Bao T	Cross-sectional	Urine	GUM clinic attendees in Tangdu	757	M	NR/NR/NR	NR
France 1	2011	Sednaoui P	Cross-sectional	Unclear	Patients attending a clinic in Paris for STI screening, a medical consultation/check-up	955	W&M	NR/NR/NR	NR
Germany 2	2015	Lallemand A	Cross-sectional	M: Urine W: Vagina	Patients seeking HIV testing in North Rhine-Westphalia	3187	W&M	NR/30/NR	NR
Great Britain 5	2014	Svenstrup HF	Cross-sectional	Cervix, vagina, urine	Women screened for CT in National Chlamydia Screening Programme and two STI clinics	4613	W	15-64/NR/NR	NR
Great Britain 6	2006	Leung A	Cross-sectional	Urethra, urine	Men attending GUM clinics in Bristol, Truro, Bath, UK	680	M	NR/NR/NR	328/680 (48%)
Great Britain 7	2014	Slack R	Cross-sectional	Urine	GUM clinic attendees in two clinics	563	M	NR/NR/NR	159/563 (28%)
Netherlands 1	2015	Van der Veer C	Cross-sectional	Urine	Men attending an STI clinic in Amsterdam	526	M	NR/37/NR	266/1204 (22%)
Norway 1	2009	Moi H	Cross-sectional	Urine	STI clinic attendees with symptoms, multiple partners, MSM, contacts of STI in Oslo	8468	M	NR/NR/31	3024/8468 (36%)
Norway 2	2009	Moi H	Cross-sectional	Urine, cervix	STI clinic attendees with symptoms, multiple partners, contacts of STI in Oslo	7646	W	NR/NR/26	NR (60-64%)
Norway 3	2011	Nilsen E	Cross-sectional	Urine, urethra or cervix	All samples sent to the Molde Hospital Laboratory, Norway, for CT testing	950	W&M	NR/NR/W26,M29	NR
Russia 1	2011	Khryanin A	Cross-sectional	Urethra and/or cervix	Patients attending antenatal clinics, hospitals, medical centers, STI clinics	9208	W&M	NR/NR/NR	NR
Russia 2	2012	Berle LM	Cross-sectional	Urine	HIV centre, STI clinic, military, students, abortion clinic	1729	W&M	NR/NR/NR	NR

Supplementary material

Country, number	Year	First author	Study type	Specimen type	Study setting and population	Sample size	Gender	Age, years range/median/mean	Number of symptomatic patients
South Korea 2	2013	Choi JY	Cross-sectional	Urine	Healthy asymptomatic policemen participating in a general prostate health checkup program in Seoul, South Korea	551	M	NR/NR/51	95/551 (17%)
Sweden 1	2003	Falk L	Cross-sectional	M: Urine<br>W: Urine, cervix	STI clinic attendees	980	W&M	NR/NR/NR	NR
Sweden 4	2005	Mellenius H	Cross-sectional	Urine	Patients tested for CT at a dermatology and STI clinic, Norrland University	823	W&M	NR/NR/NR	NR
Uganda 2	2014	Tobian AR	?	Vagina	Female partners of men in an RCT of circumcision for HIV prevention	831	W	NR/NR/NR	305/823 (37%)
USA 1	2003	Manhart LE	Cross-sectional	Cervix	Archived samples from a previous study of randomly selected STI clinic attendees	719	W	16-45/NR/NR	139/539 (26%)
USA 4	2010	Hancock EB	Cross-sectional	Vagina	Women attending an STI clinic in Seattle	1090	W	16-45/24/NR	NR
Venezuela 1	2013	Peralta-Arias RD	Cross-sectional	Cervix	First time attendees in a fertility clinic	3358	W	NR/NR/35	NR

Abbreviations: CT, Chlamydia trachomatis ; GUM, genitourinary medicine; M, men; MSM, men who have sex with men NR, not reported; STI, sexually transmitted infection; T, transgender; W, women

Supplementary material

Supplementary figures, Figure S1 to Figure S4

Figure S1. Flow chart of included and excluded studies

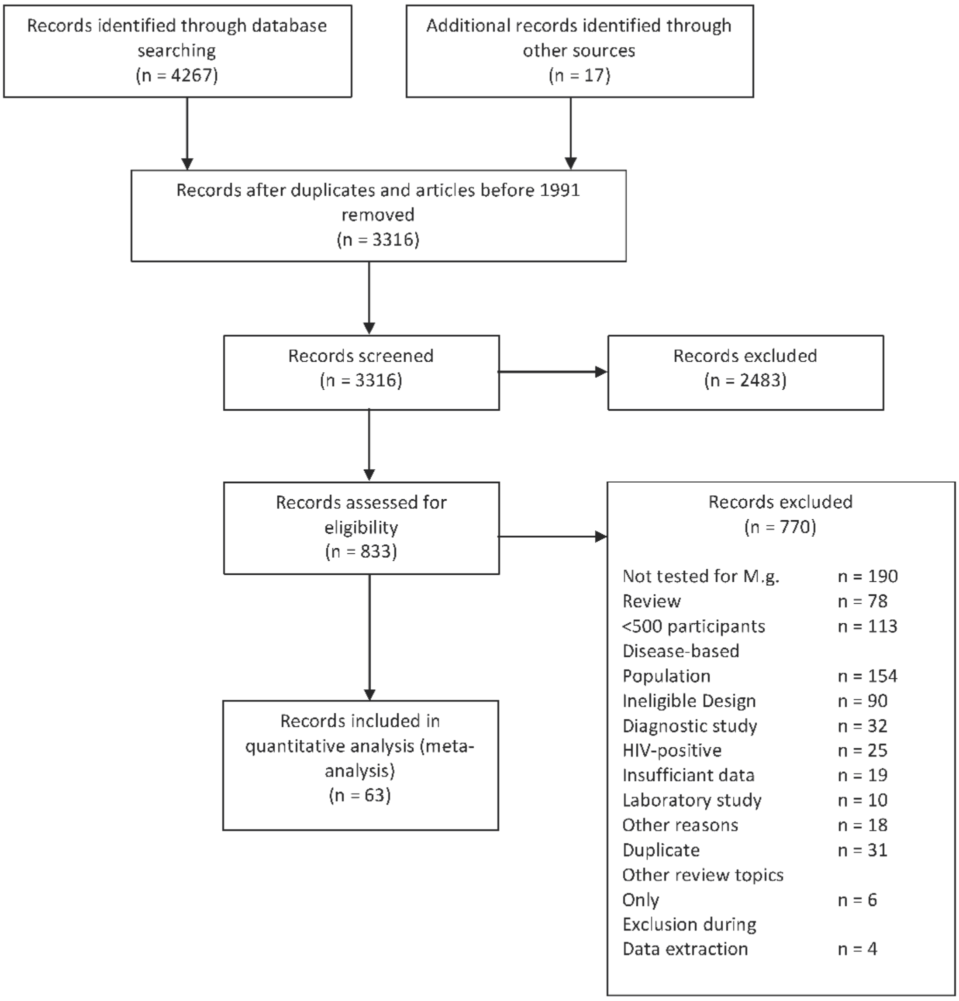

Supplementary material

Figure S2. Risk of bias assessment of included studies

Study identifier	Target population clearly defined?	Source population clearly defined?	Source population adequate sample of target population?	Similar responders and non-responders?	Probability sample used?	Sample size calculation reported?	Calculated sample size achieved?	Can we be confident in the assessment of	Data provided to calculate prevalence?	Confidence intervals reported?	Data provided on number of people asked to participate	Response rate
Australia 1, 2009	Red	Green	?	Red	Red	?	?	Green	Green	Red	Red	?
Australia 2, 2009	Green	Green	Green	Red	Red	?	?	Green	Green	Green	Green	Red
Australia 3, 2011	Green	Green	Green	Red	Red	Green	Green	Red	Green	Green	Red	Red
Australia 4, 2011	Green	Green	Red	Red	Red	?	?	Red	Green	Green	Red	Green
Benin, Ghana 1, 2005	Green	Green	Red	Red	?	?	?	Green	Green	Red	Red	?
Canada 1, 2016	Green	Green	Red	Red	Red	?	?	Green	Green	Green	Red	?
China 1, 2010	Red	Green	?	Red	Red	?	?	Red	Green	Red	Red	?
China 2, 2012	Green	Green	Green	Red	Red	?	?	Green	Green	Green	Red	?
Denmark 1, 2007	Green	Green	?	Red	Green	?	?	Green	Green	Green	Green	Red
El Salvador 1, 2012	Green	Green	?	Red	Red	Green	Green	Red	Green	Green	Red	?
France 1, 2011	Red	Green	Red	Red	Red	?	?	Red	Red	Red	Red	?
France 2, 2015	Green	Green	Green	Red	Red	Red	?	Red	Green	Green	Green	Green
France 3, 2014	Green	Green	Green	Red	Red	?	?	Green	Green	Green	Green	Green
Germany 1, 2015	Green	Green	Green	Red	?	?	?	Green	Red	Red	Red	?
Germany 2,3, 2015	Green	Green	Green	Red	Red	?	?	Red	Green	Green	Red	?
Great Britain 1, 2004	Red	Green	?	Red	Red	?	?	Green	Green	Green	Green	Yellow
Great Britain 2, 2010	Green	Green	Green	Yellow	Red	?	?	Green	Green	Green	Red	?
Great Britain 3, 2013	Red	Green	Red	Red	Red	?	?	Green	Green	Red	Red	?
Great Britain 4, 2015	Green	Green	Green	Green	Green	Green	Green	Green	Green	Green	Green	Red
Great Britain 5, 2014	Green	Green	Green	Red	Red	?	?	Red	Green	Green	Red	?
Great Britain 6, 2006	Red	Green	?	Red	Red	?	?	Green	Green	Red	Red	?
Great Britain 7, 2014	Green	Green	Green	Red	Red	?	?	Red	Green	Red	Red	?
Guatemala 1, Honduras 3, 2015	Green	Green	Green	Red	Red	?	?	Red	Green	Red	Red	?
Honduras 1, 2009	Green	Green	Green	Red	Green	Green	Green	Green	Green	Green	Green	Green
Honduras 2, 2012	Green	Green	?	Red	Red	Green	Red	Green	Green	Red	Red	?
Japan 1, 2006	Red	Green	?	Red	Red	?	?	Red	Green	Red	Red	?
Kenya 1, 2012	Red	Green	?	Yellow	?	?	?	Green	Green	Green	Red	?
Madagascar 1, 2005	Green	Green	Green	Red	?	?	?	Green	Green	Red	Red	?
Netherlands 1,2, 2015	Green	Green	Red	Red	Red	?	?	Green	Green	Red	Red	?
Nicaragua 1, 2011	Green	Green	Green	Red	Red	?	?	Red	Green	Green	Red	?
Norway 1, 2009	Green	Green	Red	Red	Red	Red	?	Green	Green	Red	Red	?
Norway 2 2009	Green	Green	Green	Red	Red	Red	?	Red	Green	Red	Red	Green
Norway 3, 2011	Red	Red	Red	Red	Red	?	?	Red	Green	Red	Red	?
Norway 4, 2013	Green	Green	Green	Red	Red	?	?	Green	Green	Red	Green	Red
Norway 5, 2013	Green	Green	Green	Red	Red	?	?	Green	Green	Red	Red	?
Norway 6, 2015	Red	Green	?	Red	Red	?	?	Green	Green	Red	Green	Green
Norway 7, 2015	Red	Red	Red	Red	Red	?	?	Red	Green	Red	Red	?
Russia 1, 2011	Green	Green	Green	Red	Red	Red	?	Red	Red	Red	Red	?
Russia 2, 2012	Green	Green	?	Red	Red	?	?	Green	Green	Red	Red	?
Russia 3, 2013	Green	Green	Green	Red	Red	?	?	Red	Green	Red	Red	?
South Africa 1, 2015	Green	Green	Green	Red	Red	?	?	Red	Green	Green	Red	?
South Korea 1, 2011	Red	Green	?	Red	Red	?	?	Green	Green	Red	Red	?

Supplementary material

Study identifier	Target population clearly defined?	Source population clearly defined?	Source population adequate sample of target population?	Similar responders and non-responders?	Probability sample used?	Sample size calculation reported?	Calculated sample size achieved?	Can we be confident in the assessment of	Data provided to calculate prevalence?	Confidence intervals reported?	Data provided on number of people asked to participate	Response rate
South Korea 2 ,2013						?	?					?
South Korea 3, 2014						?	?					?
Sweden 1, 2003			?			?	?					?
Sweden 2, 2004						?	?					
Sweden 3, 2004			?			?	?					?
Sweden 4, 2005						?	?					?
Sweden 5, 2005			?			?	?					?
Sweden 6, 2005			?			?	?					
Sweden 7, 2007			?			?	?					?
Sweden 8, 2008						?	?					
Sweden 9, 2012							?					
Tanzania 1, 2006												
Uganda 1, 2012												
Uganda 2, 2014						?	?					?
USA 1, 2003			?			?	?					?
USA 2, 2007						?	?					?
USA 3, 2008						?	?					?
USA 4, 2010			?			?	?					?
USA 5, 2014						?	?					
Venezuela 1, 2013			?			?	?					?
Vietnam 1, 2009						?	?					?

? Insufficient information to assess item

Population based, random sampling

Population based, non-random sampling

Supplementary material

Figure S3. Forest plot of studies of *M. genitalium* prevalence, by age and sex in general population samples

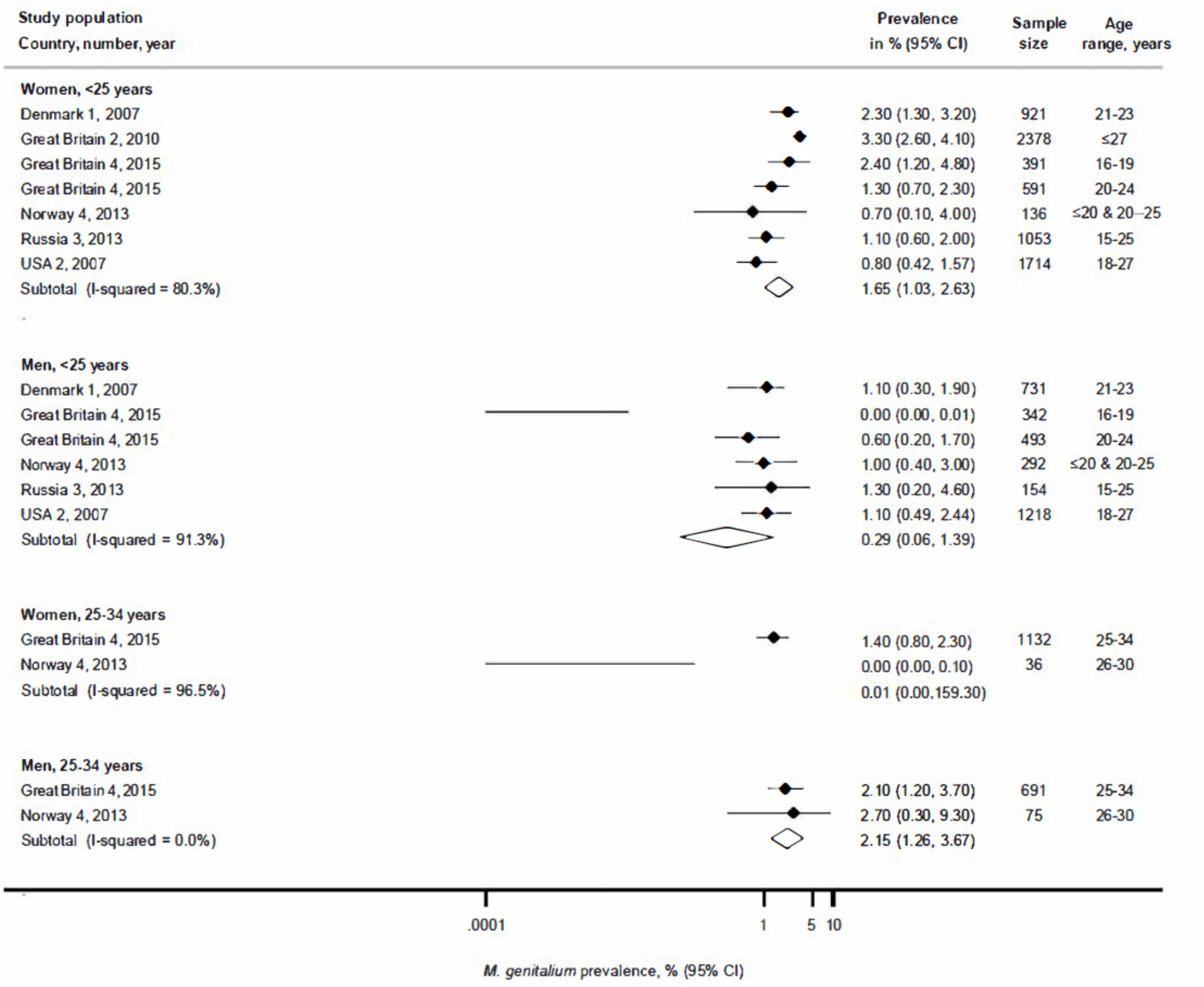

Supplementary material

Figure S4. Forest plot of studies of *M. genitalium* prevalence in healthcare clinic based settings, by symptom status of patients and enrolment process

Study population<br>Country, number, year	Prevalence<br>in % (95% CI)	Sample size<br>gender	Age<br>range or mean, years
**Asymptomatic only**			
France 3, 2014	0.60 (0.20, 1.00)	1381 W&M	<30
South Korea 1, 2011	0.30 (0.00, 1.00)	709 W&M	20-60
South Korea 3, 2014	1.00 (0.40, 2.00)	799 W	25-81
Subtotal (I-squared = 0.0%)	0.77 (0.44, 1.37)		
**Consecutive patients, with and without symptoms**			
Australia 3, 2011	2.40 (1.50, 3.30)	1116 W	16-25
Australia 4, 2011	4.00 (2.50, 6.00)	527 W	NR
Canada 1, 2016	4.20 (3.20, 5.50)	1193 W&M	19-57
Great Britain 3, 2013	1.00 (0.60, 1.60)	1718 W&M	12-87
Norway 6, 2015	6.50 (5.10, 8.10)	1097 W	NR
Norway 7, 2015	3.10 (2.90, 3.20)	78505 W&M	13-79
South Africa 1, 2015	8.70 (6.40, 10.90)	601 W	18-49
Sweden 2, 2004	7.00 (4.60, 9.20)	512 M	16-67
Sweden 3, 2004	6.80 (5.90, 7.80)	2605 W&M	NR
Sweden 5, 2005	6.10 (4.70, 7.90)	946 W&M	14-67
Sweden 6, 2005	6.40 (4.70, 8.50)	699 W&M	15-58
Sweden 7, 2007	6.60 (5.00, 8.50)	833 W&M	17-52
Sweden 8, 2008	7.40 (5.50, 9.60)	679 W&M	17-82
Sweden 9, 2012	2.10 (1.70, 2.60)	5519 W	15-46
Subtotal (I-squared = 96.1%)	4.56 (3.46, 6.00)		
**Patient enrolment not clearly described**			
Australia 1, 2009	2.80 (1.60, 4.60)	529 M	19-76
China 1, 2010	3.00 (1.90, 4.50)	757 M	NR
France 1, 2011	2.10 (1.30, 3.20)	955 W&M	NR
Germany 2, 2015	3.42 (2.80, 4.10)	3187 W&M	30
Great Britain 5, 2014	3.00 (2.50, 3.50)	4613 W	15-64
Great Britain 6, 2006	5.30 (3.70, 7.30)	680 M	NR
Great Britain 7, 2014	4.40 (2.90, 6.50)	563 M	NR
Netherlands 1, 2015	3.80 (2.30, 5.80)	526 M	37
Norway 1, 2009	3.70 (3.30, 4.10)	8468 M	31.3
Norway 2, 2009	4.00 (3.50, 4.40)	7646 W	25.5
Norway 3, 2011	2.00 (1.20, 3.10)	950 W&M	W26-M29
Russia 1, 2011	12.60 (11.90, 13.30)	9208 W&M	NR
Russia 2, 2012	3.90 (3.00, 4.90)	1729 W&M	NR
South Korea 2, 2013	1.00 (0.40, 2.40)	551 M	50.8
Sweden 1, 2003	6.10 (4.70, 7.80)	980 W&M	NR
Sweden 4, 2005	4.00 (2.80, 5.60)	823 W&M	NR
Uganda 2, 2014	3.40 (2.30, 4.80)	831 W	NR
USA 1, 2003	7.00 (5.20, 9.10)	719 W	16-45
USA 4, 2010	7.70 (6.20, 9.40)	1090 W	16-45
Venezuela 1, 2013	0.60 (0.30, 0.90)	3358 W	35.3
Subtotal (I-squared = 98.2%)	3.58 (2.56, 4.99)		

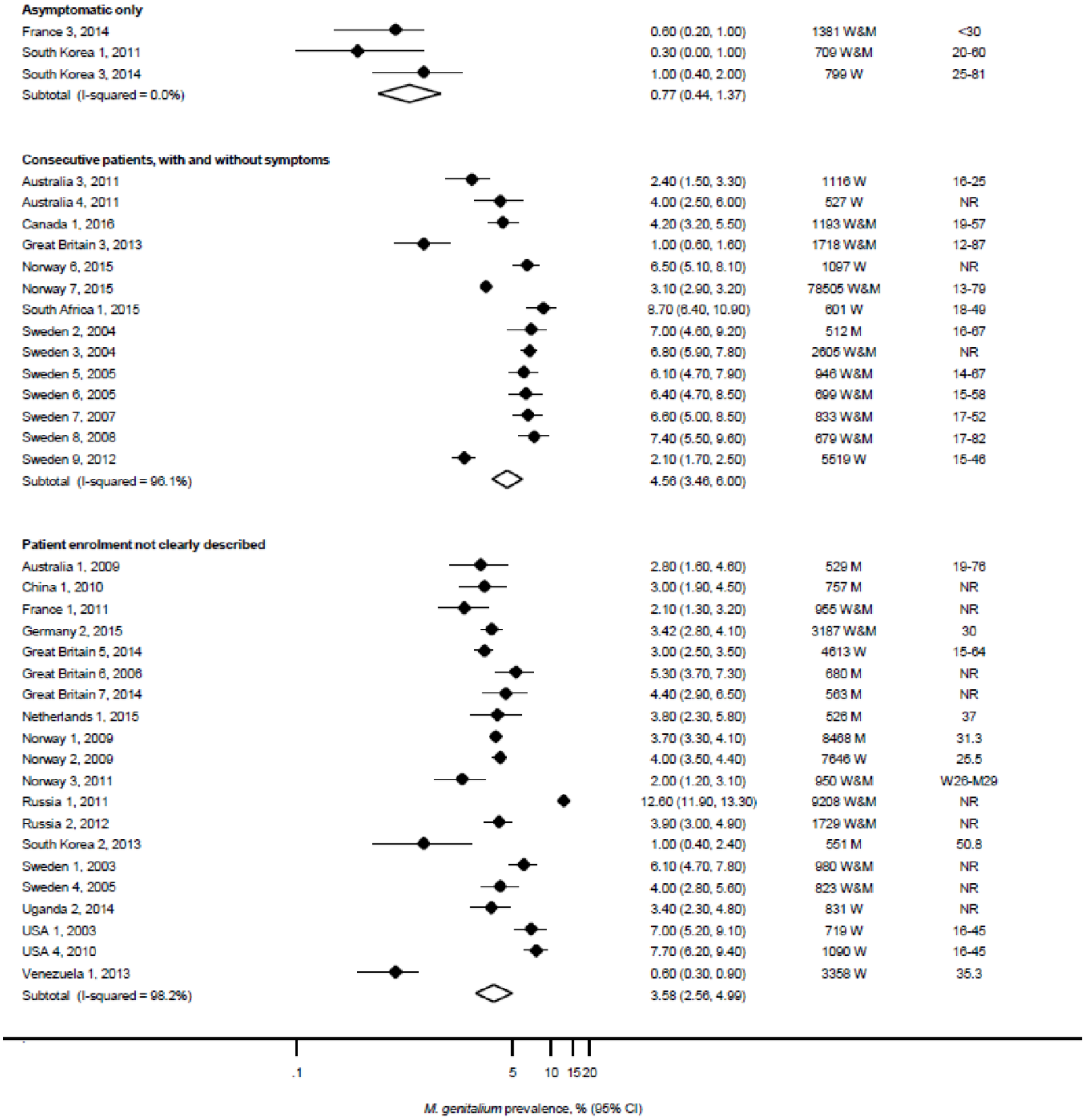

Supplement: Supplementary file 1 [file sextrans-2017-053384supp001.pdf]
