# Supplementary material for: Prevalence of Mycoplasma genitalium in different population groups: systematic review andmeta-analysis
Source: Sex Transm Infect. 2018 Feb 9;94(4):255–62. doi: 10.1136/sextrans-2017-053384 (PMC5969327; doi:10.1136/sextrans-2017-053384)
Supplement: Supplementary file 2 [file sextrans-2017-053384supp002.docx]

## Table S1. Summary characteristics of studies included in, or excluded from systematic review

| **Included** | **In_manuscript** | **First_author** | **Country_number** | **Year** | **Population** | **HDI** | **Gender** | **Publication_year** | **Country** | **Stratum** | **Number_infected** | **Number_tested** | **Percent_positive** | **Sampling_method** | **Unique_id** |
| --- | --- | --- | --- | --- | --- | --- | --- | --- | --- | --- | --- | --- | --- | --- | --- |
| 0 | Excluded | Alakija A | Not recorded | 2013 | CSW | Unclear | Women only | 2013-2016 | not reported | women | NR | 350 | 12.7 | unclear/not reported | 1 |
| 0 | Excluded | Alvarez Rodriguez B | Not recorded | 2013 | CSW | High | Women only | 2013-2016 | Belize | women | NR | 220 | 11.2 | convenience sampling | 2 |
| 0 | Excluded | Amirmozafari N | Not recorded | 2009 | Clinic | High | Women only | 2009-2012 | Iran | women | 11 | 210 | 5.2 | convenience sampling | 3 |
| 0 | Excluded | Arraiz RN | Not recorded | 2008 | Clinic | High | Women only | 2009-2012 | Venezuela | women | 13 | 172 | 7.5 | convenience sampling | 4 |
| 0 | Excluded | Averbach S H | Not recorded | 2013 | Pregnant | Very high | Women only | 2013-2016 | United States | women | NR | 95 | 8.4 | convenience sampling | 5 |
| 0 | Excluded | Baczynska A | Not recorded | 2008 | Clinic | Very high | Women only | 2005-2008 | Denmark | women | 1 | 102 | 1.0 | convenience sampling | 6 |
| 0 | Excluded | Balkus JE | Not recorded | 2016 | Clinic | Multiple countries | Women only | 2013-2016 | Kenya and United States | women | 27 | 221 | 12.2 | simple random sampling | 7 |
| 0 | Excluded | Bayette J | Not recorded | 2012 | Clinic | Very high | Women & men | 2009-2012 | France | both | 1 | 301 | 0.3 | convenience sampling | 8 |
| 0 | Excluded | Bebear C | Not recorded | 2011 | Clinic | Very high | Women & men | 2009-2012 | France | men | 5 | 269 | 1.9 | convenience sampling | 9 |
| 0 | Excluded | Bebear C | Not recorded | 2011 | Clinic | Very high | Women & men | 2009-2012 | France | women | 4 | 193 | 2.1 | convenience sampling | 9 |
| 0 | Excluded | Belokrinitskaya TE | Not recorded | 2013 | Clinic | High | Women only | 2013-2016 | Russia | women | 47 | 403 | 11.7 | convenience sampling | 10 |
| 0 | Excluded | Benning L | Not recorded | 2014 | Other | Multiple countries | Women only | 2013-2016 | Rwanda/United States | women | NR | 86 | NR | unclear/not reported | 11 |
| 0 | Excluded | Bhat PV | Not recorded | 2013 | Pregnant | Medium | Women only | 2013-2016 | India | women | 14 | 115 | 12.2 | convenience sampling | 12 |
| 0 | Excluded | Camporiondo MP | Not recorded | 2016 | Clinic | Very high | Women only | 2013-2016 | Italy | women | 0 | 309 | 0.0 | convenience sampling | 13 |
| 0 | Excluded | Chandeying V | Not recorded | 2000 | Community-based | High | Men only | ≦2000 | Thailand | men | 11 | 479 | 2.3 | consecutive sampling | 14 |
| 0 | Excluded | Chen MY | Not recorded | 2010 | CSW | Very high | Women only | 2009-2012 | Australia | women | NA | 22 | NR | convenience sampling | 15 |
| 0 | Excluded | Chiamaka Chukwuka P | Not recorded | 2013 | Community-based | Low | Women only | 2013-2016 | Nigeria | women | 6 | 100 | 6.0 | unclear/not reported | 16 |
| 0 | Excluded | Choi Y | Not recorded | 2014 | Clinic | Very high | Women only | 2013-2016 | South Korea | women | 0 | 124 | 0.0 | consecutive sampling | 17 |
| 0 | Excluded | Christofolini DM | Not recorded | 2012 | Community-based | High | Women only | 2009-2012 | Brazil | women | 1 | 106 | 9.4 | convenience sampling | 18 |
| 0 | Excluded | Cohen CR | Not recorded | 2007 | CSW | Low | Women only | 2005-2008 | Kenya | women | 40 | 255 | 15.7 | convenience sampling | 19 |
| 0 | Excluded | Cosentino LA | Not recorded | 2012 | Clinic | Very high | Women & men | 2009-2012 | United States | both | 47 | 497 | 9.5 | convenience sampling | 20 |
| 0 | Excluded | Daley GM | Not recorded | 2014 | Other | Very high | Men only | 2013-2016 | Australia | men | 8 | 140 | 5.7 | convenience sampling | 21 |
| 0 | Excluded | Datcu R | Not recorded | 2013 | Community-based | Unclear | Women only | 2013-2016 | Greenland | women | 22 | 177 | 12.0 | convenience sampling | 22 |
| 0 | Excluded | Deguchi T | Not recorded | 2009 | CSW | Very high | Women only | 2009-2012 | Japan | women | 7 | 403 | 1.7 | convenience sampling | 23 |
| 0 | Excluded | Deguchi T | Not recorded | 2015 | CSW | Very high | Women only | 2013-2016 | Japan | women | 21 | 149 | 14.1 | convenience sampling | 24 |
| 0 | Excluded | Dehon PM | Not recorded | 2014 | Clinic | Very high | Women only | 2013-2016 | United States | women | 7 | 473 | 1.5 | convenience sampling | 25 |
| 0 | Excluded | Des Marais A | Not recorded | 2014 | Unclear/not reported | Very high | Women only | 2013-2016 | United States | women | NR | 137 | 3.0 | unclear/not reported | 26 |
| 0 | Excluded | Donders GG | Not recorded | 2016 | Other | Low | Women only | 2013-2016 | Uganda | women | NR | 360 | 0.6 | consecutive sampling | 27 |
| 0 | Excluded | Downey RF | Not recorded | 2015 | Clinic | Low | Men only | 2013-2016 | Haiti | men | 13 | 205 | 6.3 | convenience sampling | 28 |
| 0 | Excluded | Dupin N | Not recorded | 2003 | Clinic | Very high | Men only | 2001-2004 | France | men | 19 | 193 | 9.8 | consecutive sampling | 29 |
| 0 | Excluded | Edberg A | Not recorded | 2009 | Clinic | Very high | Women only | 2009-2012 | Sweden | women | 25 | 329 | 7.6 | consecutive sampling | 30 |
| 0 | Excluded | Falk L | Not recorded | 2005 | Clinic | Very high | Women only | 2005-2008 | Sweden | women | 26 | 461 | 63.4 | consecutive sampling | 31 |
| 0 | Excluded | Falk L | Not recorded | 2010 | Clinic | Very high | Women only | 2009-2012 | Sweden | women | 12 | 84 | 14.3 | convenience sampling | 32 |
| 0 | Excluded | Francis SC | Not recorded | 2015 | Community-based | Low | Women only | 2013-2016 | Tanzania | women | 6 | 403 | 2.0 | convenience sampling | 33 |
| 0 | Excluded | Frolova NI | Not recorded | 2013 | Clinic | High | Women only | 2013-2016 | Russia | women | NR | 403 | 11.7 | convenience sampling | 34 |
| 0 | Excluded | Gaydos C | Not recorded | 2009 | Clinic | Very high | Women only | 2009-2012 | United States | women | 62 | 322 | 19.2 | convenience sampling | 35 |
| 0 | Excluded | Gaydos C | Not recorded | 2009 | Clinic | Very high | Men only | 2009-2012 | United States | men | NR | 290 | 15.2 | convenience sampling | 35 |
| 0 | Excluded | Gaydos CA | Not recorded | 2015 | Clinic | Unclear | Men only | 2013-2016 | unknown | men | 29 | 203 | 14.3 | convenience sampling | 36 |
| 0 | Excluded | Gesink DC | Not recorded | 2012 | Community-based | Unclear | Women & men | 2009-2012 | Greenland | both | NR | 314 | 9.8 | convenience sampling | 37 |
| 0 | Excluded | Gillespie CW | Not recorded | 2013 | Clinic | Very high | Men only | 2013-2016 | United States | men | 7 | 236 | 3.0 | consecutive sampling | 38 |
| 0 | Excluded | Gomih-Alakija A | Not recorded | 2014 | CSW | Low | Women only | 2013-2016 | Kenya | women | 45 | 349 | 12.9 | consecutive sampling | 39 |
| 0 | Excluded | Govender S | Not recorded | 2009 | Pregnant | Medium | Women only | 2009-2012 | South Africa | women | 0 | 418 | 0.0 | unclear/not reported | 40 |
| 0 | Excluded | Gubelin Harcha W | Not recorded | 2006 | Clinic | Very high | Women & men | 2005-2008 | Chile | men | 6 | 37 | 16.2 | consecutive sampling | 41 |
| 0 | Excluded | Gubelin Harcha W | Not recorded | 2006 | Clinic | Very high | Women & men | 2005-2008 | Chile | women | 6 | 40 | 15.0 | consecutive sampling | 41 |
| 0 | Excluded | Guschin A | Not recorded | 2015 | Clinic | High | Men only | 2013-2016 | Russia | men | 47 | 320 | 14.7 | consecutive sampling | 42 |
| 0 | Excluded | Haghighi Hasanabad M | Not recorded | 2011 | Pregnant | High | Women only | 2009-2012 | Iran | women | 2 | 196 | 1.0 | unclear/not reported | 43 |
| 0 | Excluded | Hahn HS | Not recorded | 2014 | Pregnant | Very high | Women only | 2013-2016 | Korea | women | NR | 455 | NR | convenience sampling | 44 |
| 0 | Excluded | Hamasuna R | Not recorded | 2008 | CSW | Very high | Women only | 2005-2008 | Japan | women | 7 | 242 | 2.8 | convenience sampling | 45 |
| 0 | Excluded | Henning D | Not recorded | 2014 | Clinic | Very high | Women & men | 2013-2016 | Australia | both | 8 | 60 | 13.3 | consecutive sampling | 46 |
| 0 | Excluded | Huppert JS | Not recorded | 2008 | Clinic | Very high | Women only | 2005-2008 | United States | women | 74 | 331 | 22.4 | convenience sampling | 47 |
| 0 | Excluded | Huppert JS | Not recorded | 2013 | Clinic | Very high | Women only | 2013-2016 | United States | women | 30 | 217 | 13.8 | convenience sampling | 48 |
| 0 | Excluded | Ito S | Not recorded | 2014 | Clinic | Very high | Men only | 2013-2016 | Japan | men | 9 | 209 | 4.3 | convenience sampling | 49 |
| 0 | Excluded | Jacobs ZC | Not recorded | 2013 | Unclear/not reported | Very high | Men only | 2013-2016 | United States | men | 6 | 75 | 8.0 | unclear/not reported | 50 |
| 0 | Excluded | Jiang J | Not recorded | 2015 | MSM | High | Men only | 2013-2016 | China | men | NR | 388 | 17.2 | convenience sampling | 51 |
| 0 | Excluded | Jobe KA | Not recorded | 2014 | Clinic | Very high | Women only | 2013-2016 | Haiti | women | 25 | 297 | 8.4 | convenience sampling | 52 |
| 0 | Excluded | Johannisson G | Not recorded | 2000 | Clinic | Very high | Women & men | ≦2000 | Sweden | men | 18 | 233 | 7.7 | unclear/not reported | 53 |
| 0 | Excluded | Johannisson G | Not recorded | 2001 | Clinic | Very high | Women & men | 2001-2004 | Sweden | women | 3 | 85 | 3.5 | unclear/not reported | 54 |
| 0 | Excluded | Jombo GTA | Not recorded | 2009 | Pregnant | Low | Women only | 2009-2012 | Nigeria | women | NA | 283 | NR | consecutive sampling | 55 |
| 0 | Excluded | Justel M | Not recorded | 2015 | Pregnant | Low | Women only | 2013-2016 | Angola | women | 19 | 312 | 6.1 | convenience sampling | 56 |
| 0 | Excluded | Keane FE | Not recorded | 2000 | Clinic | Very high | Women & men | ≦2000 | United Kingdom | both | 9 | 76 | 11.8 | unclear/not reported | 57 |
| 0 | Excluded | Khryanin AA | Not recorded | 2012 | Unclear/not reported | High | Unknown | 2009-2012 | Russia | NR | NR | 75 | NR | unclear/not reported | 58 |
| 0 | Excluded | Kim TH | Not recorded | 2007 | Unclear/not reported | Very high | Men only | 2005-2008 | Korea | men | NA | NA | NA | unclear/not reported | 59 |
| 0 | Excluded | Kjaergaard N | Not recorded | 1997 | Clinic | Very high | Men only | ≦2000 | Denmark | men | 5 | 187 | 0.9 | unclear/not reported | 60 |
| 0 | Excluded | Kwatampora J | Not recorded | 2011 | CSW | Unclear | Women only | 2009-2012 | Nairobi | women | NA | 299 | 20.7 | convenience sampling | 61 |
| 0 | Excluded | Lawton BA | Not recorded | 2008 | Pregnant | Very high | Women only | 2005-2008 | New Zealand | women | 26 | 300 | 8.7 | convenience sampling | 62 |
| 0 | Excluded | Le Roy C | Not recorded | 2012 | Clinic | Very high | Women & men | 2009-2012 | France | both | 9 | 453 | 2.0 | consecutive sampling | 63 |
| 0 | Excluded | Lee J | Not recorded | 2016 | Other | Very high | Women & men | 2013-2016 | Korea | both | NR | 237 | 4.2 | unclear/not reported | 64 |
| 0 | Excluded | Lewis DA | Not recorded | 2008 | Clinic | Medium | Men only | 2005-2008 | South Africa | men | NR | 301 | NR | convenience sampling | 65 |
| 0 | Excluded | Lillis RA | Not recorded | 2011 | Clinic | Very high | Women only | 2009-2012 | United States | women | 70 | 400 | 17.5 | convenience sampling | 66 |
| 0 | Excluded | Liu CM | Not recorded | 2014 | MSM | Very high | Men only | 2013-2016 | United States | men | NR | 49 | NR | unclear/not reported | 67 |
| 0 | Excluded | Ljubin-Sternak S | Not recorded | 2014 | Unclear/not reported | Very high | Women & men | 2013-2016 | Croatia | both | 6 | 195 | 3.1 | convenience sampling | 68 |
| 0 | Excluded | Magaña-Contreras M | Not recorded | 2015 | Clinic | High | Women only | 2013-2016 | Mexico | women | 1 | 201 | 0.5 | convenience sampling | 69 |
| 0 | Excluded | Mahmutovic-Vranic S | Not recorded | 2007 | Clinic | High | Women only | 2005-2008 | Bosnia and Herzegovina | women | 14 | 92 | 14.4 | convenience sampling | 70 |
| 0 | Excluded | Masson L | Not recorded | 2015 | Community-based | Medium | Women only | 2013-2016 | South Africa | women | 3 | 227 | 1.3 | convenience sampling | 71 |
| 0 | Excluded | Mawu FO | Not recorded | 2011 | CSW | Medium | Women only | 2009-2012 | Indonesia | women | 27 | 217 | 12.4 | convenience sampling | 72 |
| 0 | Excluded | McIver CJ | Not recorded | 2009 | Clinic | Very high | Women only | 2009-2012 | Australia | women | 3 | 175 | 1.7 | consecutive sampling | 73 |
| 0 | Excluded | McKechnie ML | Not recorded | 2011 | Clinic | Very high | Women only | 2009-2012 | Australia | women | 10 | 216 | 4.6 | consecutive sampling | 74 |
| 0 | Excluded | Mihalik N | Not recorded | 2013 | Clinic | Very high | Women & men | 2013-2016 | Hungary | both | 1 | 51 | 2.0 | unclear/not reported | 75 |
| 0 | Excluded | Mobley VL | Not recorded | 2012 | Clinic | Very high | Women only | 2009-2012 | United States | women | 73 | 381 | 19.2 | convenience sampling | 76 |
| 0 | Excluded | Morales-Miranda S | Not recorded | 2013 | MSM | High | Men only | 2013-2016 | Belize | men | NR | 130 | 2.8 | convenience sampling | 77 |
| 0 | Excluded | Muzny CA | Not recorded | 2011 | Clinic | Very high | Women only | 2009-2012 | United States | women | 14 | 191 | 7.6 | consecutive sampling | 78 |
| 0 | Excluded | Muzny CA | Not recorded | 2014 | Clinic | Very high | Women only | 2013-2016 | United Kingdom | women | 12 | 163 | 8.0 | convenience sampling | 79 |
| 0 | Excluded | Nakashima K | Not recorded | 2014 | Clinic | Very high | Men only | 2013-2016 | Japan | men | 16 | 213 | 7.7 | convenience sampling | 80 |
| 0 | Excluded | Nelson A | Not recorded | 2007 | Clinic | High | Women & men | 2005-2008 | Peru | men | 3 | 195 | 1.5 | convenience sampling | 81 |
| 0 | Excluded | Nelson A | Not recorded | 2007 | Clinic | High | Women & men | 2005-2008 | Peru | women | 7 | 195 | 3.6 | convenience sampling | 81 |
| 0 | Excluded | Olinger GG | Not recorded | 1999 | Clinic | Very high | Women only | ≦2000 | United States | women | 0 | 17 | 0.0 | convenience sampling | 82 |
| 0 | Excluded | Oliphant J | Not recorded | 2013 | Clinic | Very high | Women only | 2013-2016 | New Zealand | women | 22 | 261 | 8.4 | consecutive sampling | 83 |
| 0 | Excluded | Oliphant J | Not recorded | 2016 | Clinic | Very high | Women only | 2013-2016 | New Zealand | women | 22 | 261 | 8.4 | consecutive sampling | 83 |
| 0 | Excluded | Onodera S | Not recorded | 2012 | Clinic | Very high | Men only | 2009-2012 | Japan | men | NR | 22 | NR | unclear/not reported | 84 |
| 0 | Excluded | Ouzounova-Raykova VV | Not recorded | 2011 | Unclear/not reported | High | Women only | 2009-2012 | Bulgaria | women | NR | 348 | 0.3 | unclear/not reported | 85 |
| 0 | Excluded | Pagani C | Not recorded | 2012 | Clinic | Very high | Women only | 2009-2012 | Italy | women | 2 | 233 | 0.8 | unclear/not reported | 86 |
| 0 | Excluded | Palmer HM | Not recorded | 1991 | Clinic | Very high | Women only | ≦2000 | United Kingdom | women | 10 | 57 | 17.5 | convenience sampling | 87 |
| 0 | Excluded | Patel S | Not recorded | 2011 | Clinic | Very high | Men only | 2009-2012 | United Kingdom | men | 12 | 129 | 9.3 | unclear/not reported | 88 |
| 0 | Excluded | Peipert J | Not recorded | 2014 | Clinic | Very high | Women only | 2013-2016 | United States | women | NR | 262 | 12.5 | unclear/not reported | 89 |
| 0 | Excluded | Plaas K | Not recorded | 2015 | MSM | Very high | Men only | 2013-2016 | Estonia | men | NR | 233 | 2.6 | convenience sampling | 90 |
| 0 | Excluded | Plamenova GM | Not recorded | 2015 | Unclear/not reported | High | Women only | 2013-2016 | Bulgaria | women | NR | 135 | 0.7 | unclear/not reported | 91 |
| 0 | Excluded | Rahman S | Not recorded | 2008 | Clinic | Medium | Women only | 2005-2008 | Bangladesh | women | 3 | 399 | 0.8 | convenience sampling | 92 |
| 0 | Excluded | Rakovskaia IV | Not recorded | 2013 | Other | High | Men only | 2013-2016 | Russia | men | NR | 5 | NR | convenience sampling | 93 |
| 0 | Excluded | Redelinghuys MJ | Not recorded | 2013 | Pregnant | Medium | Women only | 2013-2016 | South Africa | women | 32 | 221 | 14.5 | unclear/not reported | 94 |
| 0 | Excluded | Redelinghuys MJ | Not recorded | 2015 | Pregnant | Medium | Women only | 2013-2016 | South Africa | women | 33 | 220 | 15.0 | unclear/not reported | 94 |
| 0 | Excluded | Rimoldi SG | Not recorded | 2012 | Clinic | Very high | Women & men | 2009-2012 | Italy | both | NR | 57 | 1.7 | convenience sampling | 95 |
| 0 | Excluded | Ross JD | Not recorded | 2009 | Clinic | Very high | Women & men | 2009-2012 | United Kingdom | both | 14 | 306 | 4.6 | convenience sampling | 96 |
| 0 | Excluded | Sellami H | Not recorded | 2014 | Clinic | High | Men only | 2013-2016 | Tunisia | men | 3 | 85 | 3.5 | unclear/not reported | 97 |
| 0 | Excluded | Shehabi AA | Not recorded | 2009 | Clinic | High | Women & men | 2009-2012 | Jordan | both | NR | 383 | NR | convenience sampling | 98 |
| 0 | Excluded | Shim HS | Not recorded | 2010 | Clinic | Very high | Women only | 2009-2012 | South Korea | women | NR | 235 | NR | convenience sampling | 99 |
| 0 | Excluded | Shipitsyna E | Not recorded | 2013 | Clinic | High | Women & men | 2013-2016 | Russia | both | NR | 432 | 4.6 | consecutive sampling | 100 |
| 0 | Excluded | Soni S | Not recorded | 2010 | MSM | Very high | Men only | 2009-2012 | United Kingdom | men | 31 | 438 | 7.1 | convenience sampling | 101 |
| 0 | Excluded | Summerton J | Not recorded | 2007 | Clinic | Very high | Men only | 2005-2008 | United States | men | 40 | 279 | 14.3 | convenience sampling | 102 |
| 0 | Excluded | Svenstrup HF | Not recorded | 2005 | Clinic | Very high | Men only | 2005-2008 | Sweden | men | 58 | 205 | 28.2 | convenience sampling | 103 |
| 0 | Excluded | Svenstrup HF | Not recorded | 2006 | Clinic | Very high | Men only | 2005-2008 | Denmark | men | 17 | 99 | 17.2 | unclear/not reported | 104 |
| 0 | Excluded | Takahashi S | Not recorded | 2006 | Community-based | Very high | Men only | 2005-2008 | Japan | men | 1 | 100 | 1.0 | convenience sampling | 105 |
| 0 | Excluded | Taylor-Robinson D | Not recorded | 2009 | Clinic | High | Men only | 2009-2012 | Russia | men | 45 | 172 | 26.2 | unclear/not reported | 106 |
| 0 | Excluded | Ting J | Not recorded | 2013 | CSW | Low | Women only | 2013-2016 | Kenya | women | 44 | 344 | 12.8 | convenience sampling | 107 |
| 0 | Excluded | Uno M | Not recorded | 1997 | Clinic | Very high | Men only | ≦2000 | Japan | men | 2 | 187 | 1.1 | unclear/not reported | 108 |
| 0 | Excluded | Williams JA | Not recorded | 2013 | Unclear/not reported | Very high | Men only | 2013-2016 | United States | men | 6 | 75 | 8.0 | unclear/not reported | 109 |
| 0 | Excluded | Worm AM | Not recorded | 1997 | Other | Very high | Men only | ≦2000 | Denmark | men | 1 | 122 | 0.8 | convenience sampling | 110 |
| 0 | Excluded | Yin Y | Not recorded | 2013 | Clinic | High | Men only | 2013-2016 | China | men | 114 | 423 | 28.1 | convenience sampling | 111 |
| 0 | Excluded | Yu JT | Not recorded | 2008 | Clinic | High | Men only | 2005-2008 | China | men | 15 | 334 | 4.5 | consecutive sampling | 112 |
| 0 | Excluded | Zheng BJ | Not recorded | 2014 | MSM | High | Men only | 2013-2016 | China | men | 33 | 406 | 8.1 | convenience sampling | 113 |
| 1 | Table S5 | Clarivet B | France 3 | 2014 | Clinic | Very high | Women & men | 2013-2016 | France | both | 8 | 1381 | 0.6 | consecutive sampling | 114 |
| 1 | Table S5 | Kim SJ | South Korea 1 | 2011 | Clinic | Very high | Women & men | 2009-2012 | South Korea | both | 2 | 709 | 0.3 | unclear/not reported | 115 |
| 1 | Table S5 | Kim Y | South Korea 3 | 2014 | Clinic | Very high | Women only | 2013-2016 | South Korea | women | 8 | 799 | 1.0 | convenience sampling | 116 |
| 1 | Table S5 | Walker J | Australia 3 | 2011 | Clinic | Very high | Women only | 2009-2012 | Australia | women | 27 | 1116 | 2.4 | consecutive sampling | 117 |
| 1 | Table S5 | Lusk MJ | Australia 4 | 2011 | Clinic | Very high | Women only | 2009-2012 | Australia | women | 21 | 527 | 4.0 | consecutive sampling | 118 |
| 1 | Table S5 | Gesink D | Canada 1 | 2016 | Clinic | Very high | Women & men | 2013-2016 | Canada | both | 50 | 1193 | 4.2 | consecutive sampling | 119 |
| 1 | Table S5 | Jalal H | Great Britain 3 | 2013 | Clinic | Very high | Women & men | 2013-2016 | United Kingdom | both | 17 | 1718 | 1.0 | consecutive sampling | 120 |
| 1 | Table S5 | Hartgill U | Norway 6 | 2015 | Clinic | Very high | Women only | 2013-2016 | Norway | women | 71 | 1097 | 6.5 | consecutive sampling | 121 |
| 1 | Table S5 | Reinton N | Norway 7 | 2015 | Clinic | Very high | Women & men | 2013-2016 | Norway | both | 2398 | 78505 | 3.1 | convenience sampling | 122 |
| 1 | Table S5 | Hay B | South Africa 1 | 2015 | Clinic | Medium | Women only | 2013-2016 | South Africa | women | 52 | 601 | 8.7 | consecutive sampling | 123 |
| 1 | Table S5 | Falk L | Sweden 2 | 2004 | Clinic | Very high | Men only | 2001-2004 | Sweden | men | 34 | 512 | 7.0 | consecutive sampling | 124 |
| 1 | Table S5 | Jensen JS | Sweden 3 | 2004 | Clinic | Very high | Women & men | 2001-2004 | Sweden | both | 177 | 2605 | 6.8 | consecutive sampling | 125 |
| 1 | Table S5 | Anagrius C | Sweden 5 | 2005 | Clinic | Very high | Women & men | 2005-2008 | Sweden | both | 58 | 946 | 6.1 | consecutive sampling | 126 |
| 1 | Table S5 | Jurstrand M | Sweden 6 | 2005 | Clinic | Very high | Women & men | 2005-2008 | Sweden | both | 45 | 699 | 6.4 | consecutive sampling | 127 |
| 1 | Table S5 | Hogdahl M | Sweden 7 | 2007 | Clinic | Very high | Women & men | 2005-2008 | Sweden | both | 55 | 833 | 6.6 | consecutive sampling | 128 |
| 1 | Table S5 | Edberg A | Sweden 8 | 2008 | Clinic | Very high | Women & men | 2005-2008 | Sweden | both | 50 | 679 | 7.4 | consecutive sampling | 129 |
| 1 | Table S5 | Bjartling C | Sweden 9 | 2012 | Clinic | Very high | Women only | 2009-2012 | Sweden | women | 116 | 5519 | 2.1 | consecutive sampling | 130 |
| 1 | Table S5 | McKechnie ML | Australia 1 | 2009 | Clinic | Very high | Men only | 2009-2012 | Australia | men | 15 | 529 | 2.8 | unclear/not reported | 131 |
| 1 | Table S5 | Bao T | China 1 | 2010 | Clinic | High | Men only | 2009-2012 | China | men | 23 | 757 | 3.0 | unclear/not reported | 132 |
| 1 | Table S5 | Sednaoui P | France 1 | 2011 | Clinic | Very high | Women & men | 2009-2012 | France | both | 20 | 955 | 2.1 | convenience sampling | 133 |
| 1 | Table S5 | Lallemand A | Germany 2 | 2015 | Clinic | Very high | Women & men | 2013-2016 | Germany | both | 109 | 3187 | 3.4 | unclear/not reported | 134 |
| 1 | Table S5 | Svenstrup HF | Great Britain 5 | 2014 | Clinic | Very high | Women only | 2013-2016 | United Kingdom | women | 138 | 4613 | 3.0 | convenience sampling | 135 |
| 1 | Table S5 | Leung A | Great Britain 6 | 2006 | Clinic | Very high | Men only | 2005-2008 | United Kingdom | men | 36 | 680 | 5.3 | convenience sampling | 136 |
| 1 | Table S5 | Slack R | Great Britain 7 | 2014 | Clinic | Very high | Men only | 2013-2016 | United Kingdom | men | 25 | 563 | 4.4 | convenience sampling | 137 |
| 1 | Table S5 | Van der Veer C | Netherlands 1 | 2015 | Clinic | Very high | Men only | 2013-2016 | Netherlands | men | 20 | 526 | 3.8 | convenience sampling | 138 |
| 1 | Table S5 | Moi H | Norway 1 | 2009 | Clinic | Very high | Men only | 2009-2012 | Norway | men | 314 | 8468 | 3.7 | consecutive sampling | 139 |
| 1 | Table S5 | Moi H | Norway 2 | 2009 | Clinic | Very high | Women only | 2009-2012 | Norway | women | 304 | 7646 | 4.0 | consecutive sampling | 140 |
| 1 | Table S5 | Nilsen E | Norway 3 | 2011 | Clinic | Very high | Women & men | 2009-2012 | Norway | both | 19 | 950 | 2.0 | convenience sampling | 141 |
| 1 | Table S5 | Khryanin A | Russia 1 | 2011 | Clinic | High | Women & men | 2009-2012 | Russia | both | 1160 | 9208 | 12.6 | unclear/not reported | 142 |
| 1 | Table S5 | Berle LM | Russia 2 | 2012 | Clinic | High | Women & men | 2009-2012 | Russia | both | 67 | 1729 | 3.9 | unclear/not reported | 143 |
| 1 | Table S5 | Choi JY | South Korea 2 | 2013 | Clinic | Very high | Men only | 2013-2016 | South Korea | men | 6 | 551 | 1.0 | unclear/not reported | 144 |
| 1 | Table S5 | Falk L | Sweden 1 | 2003 | Clinic | Very high | Women & men | 2001-2004 | Sweden | both | 60 | 980 | 6.1 | unclear/not reported | 145 |
| 1 | Table S5 | Mellenius H | Sweden 4 | 2005 | Clinic | Very high | Women & men | 2005-2008 | Sweden | both | 33 | 823 | 4.0 | convenience sampling | 146 |
| 1 | Table S5 | Tobian AR | Uganda 2 | 2014 | Clinic | Low | Women only | 2013-2016 | Uganda | women | 28 | 831 | 3.4 | convenience sampling | 147 |
| 1 | Table S5 | Manhart LE | USA 1 | 2003 | Clinic | Very high | Women only | 2001-2004 | United States | women | 50 | 719 | 7.0 | convenience sampling | 148 |
| 1 | Table S5 | Hancock EB | USA 4 | 2010 | Clinic | Very high | Women only | 2009-2012 | United States | women | 84 | 1090 | 7.7 | unclear/not reported | 149 |
| 1 | Table S5 | Peralta-Arias RD | Venezuela 1 | 2013 | Clinic | High | Women only | 2013-2016 | Venezuela | women | 19 | 3358 | 0.6 | convenience sampling | 150 |
| 1 | Table S4 | Bradshaw CS | Australia 2 | 2009 | MSM | Very high | Men only | 2009-2012 | Australia | men | 11 | 510 | 2.1 | convenience sampling | 151 |
| 1 | Table S4 | Creswell J | El Salvador 1 | 2012 | MSM | Medium | Men only | 2009-2012 | El Salvador | men | 16 | 647 | 2.5 | snowball sampling | 152 |
| 1 | Table S4 | Ham D | Guatemala 1 | 2015 | MSM | Medium | Men only | 2013-2016 | Guatemala | men | 30 | 524 | 5.7 | snowball sampling | 153 |
| 1 | Table S4 | Ham D | Honduras 3 | 2015 | MSM | Medium | Men only | 2013-2016 | Honduras | men | 34 | 688 | 4.9 | snowball sampling | 153 |
| 1 | Table S4 | Hernandez F | Nicaragua 1 | 2011 | MSM | Medium | Men only | 2009-2012 | Nicaragua | men | 16 | 643 | 2.4 | snowball sampling | 154 |
| 1 | Table S4 | Lallemand A | Germany 3 | 2015 | MSM | Very high | Men only | 2013-2016 | Germany | men | 11 | 549 | 2.0 | unclear/not reported | 134 |
| 1 | Table S4 | Van der Veer C | Netherlands 2 | 2015 | MSM | Very high | Men only | 2013-2016 | Netherlands | men | 17 | 678 | 2.5 | convenience sampling | 138 |
| 1 | Table S4 | Reinton N | Norway 5 | 2013 | MSM | Very high | Men only | 2013-2016 | Norway | men | 91 | 1778 | 5.1 | consecutive sampling | 155 |
| 1 | Table S4 | Francis SC | USA 3 | 2008 | MSM | Very high | Men only | 2005-2008 | United States | men | 27 | 500 | 5.4 | consecutive sampling | 156 |
| 1 | Table S4 | Xiang Z | China 2 | 2012 | CSW | High | Women only | 2009-2012 | China | women | 107 | 810 | 13.2 | quota sampling | 157 |
| 1 | Table S4 | Jansen K | Germany 1 | 2015 | CSW | Very high | Women only | 2013-2016 | Germany | women | 266 | 1445 | 18.4 | unclear/not reported | 158 |
| 1 | Table S4 | Johnston LG | Honduras 2 | 2012 | CSW | Medium | Women only | 2009-2012 | Honduras | women | 133 | 726 | 18.3 | snowball sampling | 159 |
| 1 | Table S4 | Vandepitte J | Uganda 1 | 2012 | CSW | Low | Women only | 2009-2012 | Uganda | women | 148 | 1025 | 14.0 | convenience sampling | 160 |
| 1 | Table S4 | Pepin J | Benin, Ghana 1 | 2005 | CSW | Mixed | Women only | 2005-2008 | Benin, Ghana | women | 217 | 826 | 26.3 | unclear/not reported | 161 |
| 1 | Table S4 | Peuchant O | France 2 | 2015 | Pregnant | Very high | Women only | 2013-2016 | France | women | 8 | 1004 | 0.8 | convenience sampling | 162 |
| 1 | Table S4 | Oakeshott P | Great Britain 1 | 2004 | Pregnant | Very high | Women only | 2001-2004 | United Kingdom | women | 6 | 915 | 0.7 | consecutive sampling | 163 |
| 1 | Table S4 | Kataoka S | Japan 1 | 2006 | Pregnant | Very high | Women only | 2005-2008 | Japan | women | 7 | 877 | 0.8 | unclear/not reported | 164 |
| 1 | Table S4 | Agger WA | USA 5 | 2014 | Pregnant | Very high | Women only | 2013-2016 | United States | women | 9 | 676 | 1.3 | unclear/not reported | 165 |
| 1 | Table S2 | Andersen B | Denmark 1 | 2007 | General population | Very high | Women & men | 2005-2008 | Denmark | both | 29 | 1652 | 1.8 | simple random sampling | 166 |
| 1 | Table S2 | Sonnenberg P | Great Britain 4 | 2015 | General population | Very high | Women & men | 2013-2016 | Great Britain | both | 72 | 4507 | 1.2 | stratified random sampling | 167 |
| 1 | Table S2 | Manhart LE | USA 2 | 2007 | General population | Very high | Women & men | 2005-2008 | USA | both | 33 | 2932 | 1.0 | simple random sampling | 168 |
| 1 | Table S2 | Paz-Bailey G | Honduras 1 | 2009 | General population | Medium | Women & men | 2009-2012 | Honduras | both | 47 | 791 | 7.1 | stratified random sampling | 169 |
| 1 | Table S2 | Kapiga SH | Tanzania 1 | 2006 | General population | Low | Women & men | 2005-2008 | Tanzania | both | 76 | 2028 | 3.7 | stratified random sampling | 170 |
| 1 | Table S2 | Olsen B | Vietnam 1 | 2009 | General population | Medium | Women only | 2009-2012 | Vietnam | women | 8 | 990 | 0.8 | stratified random sampling | 171 |
| 1 | Table S2 | Oakeshott P | Great Britain 2 | 2010 | Community-based | Very high | Women only | 2009-2012 | Great Britain | women | 78 | 2378 | 3.3 | convenience sampling | 172 |
| 1 | Table S2 | Jensen AJ | Norway 4 | 2013 | Community-based | Very high | Women & men | 2013-2016 | Norway | both | 6 | 655 | 0.9 | unclear/not reported | 173 |
| 1 | Table S2 | Shipitsyna E | Russia 3 | 2013 | Community-based | High | Women & men | 2013-2016 | Russia | both | 14 | 1207 | 1.2 | consecutive sampling | 174 |
| 1 | Table S2 | Mehta SD | Kenya 1 | 2012 | Community-based | Low | Men only | 2009-2012 | Kenya | men | 52 | 526 | 9.9 | convenience sampling | 175 |
| 1 | Table S2 | Leutscher PDC | Madagascar 1 | 2005 | Community-based | Low | Women & men | 2005-2008 | Madagascar | both | 23 | 643 | 3.6 | unclear/not reported | 176 |

CSW, commercial sex workers; MSM, Men who have sex with men
